# Simulating short-term light responses of photosynthesis and water use efficiency in sweet sorghum under varying temperature and CO_2_ conditions

**DOI:** 10.3389/fpls.2024.1291630

**Published:** 2024-03-28

**Authors:** Xiao-Long Yang, Xiao-Fei Ma, Zi-Piao Ye, Long-Sheng Yang, Jun-Bo Shi, Xun Wang, Bei-Bei Zhou, Fu-Biao Wang, Zi-Fa Deng

**Affiliations:** ^1^ School of Life Sciences, Nantong University, Nantong, China; ^2^ State Key Laboratory of Environmental Chemistry and Ecotoxicology, Research Center for Eco-Environmental Sciences, Chinese Academy of Sciences, Beijing, China; ^3^ Key Laboratory of Ecological Safety and Sustainable Development in Arid Lands, Northwest Institute of Eco-Environment and Resources, Chinese Academy of Sciences, Lanzhou, China; ^4^ Institute of Biophysics in Maths & Physics College, Jinggangshan University, Ji’an, China

**Keywords:** sweet sorghum, leaf gas exchange, photosynthetic light response, intrinsic water use efficiency, instantaneous water use efficiency, model, temperature, CO2 concentration

## Abstract

Climate change, characterized by rising atmospheric CO_2_ levels and temperatures, poses significant challenges to global crop production. Sweet sorghum, a prominent C_4_ cereal extensively grown in arid areas, emerges as a promising candidate for sustainable bioenergy production. This study investigated the responses of photosynthesis and leaf-scale water use efficiency (WUE) to varying light intensity (*I*) in sweet sorghum under different temperature and CO_2_ conditions. Comparative analyses were conducted between the *A*_n_-*I*, *g*_s_-*I*, *T*_r_-*I*, WUE_i_-*I*, and WUE_inst_-*I* models proposed by Ye et al. and the widely utilized the non-rectangular hyperbolic (NRH) model for fitting light response curves. The Ye's models effectively replicated the light response curves of sweet sorghum, accurately capturing the diminishing intrinsic WUE (WUE_i_) and instantaneous WUE (WUE_inst_) trends with increasing *I*. The fitted maximum values of *A*_n_, *g*_s_, *T*_r_, WUE_i_, and WUE_inst_ and their saturation light intensities closely matched observations, unlike the NRH model. Despite the NRH model demonstrating high *R* ^2^ values for *A*_n_-*I*, *g*_s_-*I*, and *T*_r_-*I* modelling, it returned the maximum values significantly deviating from observed values and failed to generate saturation light intensities. It also inadequately represented WUE responses to *I*, overestimating WUE. Across different leaf temperatures, *A*_n_, *g*_s_, and *T*_r_ of sweet sorghum displayed comparable light response patterns. Elevated temperatures increased maximum *A*_n_, *g*_s_, and *T*_r_ but consistently declined maximum WUE_i_ and WUE_inst_. However, WUE_inst_ declined more sharply due to the disproportionate transpiration increase over carbon assimilation. Critically, sweet sorghum *A*_n_ saturated at current atmospheric CO_2_ levels, with no significant gains under 550 μmol mol^−1^. Instead, stomatal closure enhanced WUE under elevated CO_2_ by coordinated *g*_s_ and *T*_r_ reductions rather than improved carbon assimilation. Nonetheless, this response diminished under simultaneously high temperature, suggesting intricate interplay between CO_2_ and temperature in modulating plant responses. These findings provide valuable insights into photosynthetic dynamics of sweet sorghum, aiding predictions of yield and optimization of cultivation practices. Moreover, our methodology serves as a valuable reference for evaluating leaf photosynthesis and WUE dynamics in diverse plant species.

## Introduction

Water plays a crucial role in maintaining the structural integrity and functionality of plants, facilitating nutrient transport, and regulating temperature (Fracasso et al., 2017; Bykova et al., 2019). Specially, it is essential for photosynthesis, providing the electrons necessary to fuel the light-dependent reactions of the process. Due to the growing population, uncontrolled urbanization, and industrialization, atmospheric CO_2_ concentration has markedly risen from a pre-industrial value of 280 to 414 μmol mol^−1^ in 2020, and it is projected to exceed 700 μmol mol^−1^ by the end of 21st century (Asadi and Eshghizadeh, 2021). If the increase in CO_2_ continues, global mean temperatures are expected to rise by 2.6~4.8°C (Hatfield and Dold, 2019). Consequently, alterations in precipitation patterns have been observed in numerous regions in recent years, leading to the frequent occurrence of extreme events such as recurrent droughts and durative high temperatures. This phenomenon is particularly pronounced in arid and semi-arid areas where water resources are significantly stressed (Grillakis, 2019; Feng et al., 2022). As a consequence, crop production in these regions has been significantly limited by availability of water (Kubien et al., 2003; Prasad et al., 2009; Scafaro et al., 2011; Soltani et al., 2023).

Sweet sorghum [*Sorghum bicolor* (L.) Moench], a C_4_ species with high photosynthetic efficiency, ranks as the fifth most cereal crop globally, with the total yield lower than that of wheat, rice, maize, and barley (Loreto et al., 1995; Prasad et al., 2009; Asadi and Eshghizadeh, 2021). Due to its excellent drought tolerance, sweet sorghum is extensively cultivated in arid and semi-arid regions with annual precipitation of only 350~700 mm (Appiah-Nkansah et al., 2019; Asadi and Eshghizadeh, 2021). In 2019, global sorghum production reached 56.7 million tons, with Asia contributing 13%, and cultivation concentrated primarily in India and China (Mundia et al., 2019). Sweet sorghum can be utilized for various purposes, including starchy grains for human food, soluble sugar juice as a feedstock for producing spirits, syrup, and vinegar, and lignocellulosic biomass for manufacturing animal feed, fuel ethanol, and fiber. The crop is generating significant interest in industrial circles as a leading candidate for bioenergy production, given the remarkable accumulation of high sugar content in its stems (9~15% of dry biomass) (Appiah-Nkansah et al., 2019; Asadi and Eshghizadeh, 2021).

Photosynthesis stands as one of the pivotal life processes for plants, serving as the foundation for their growth and development. Crop yield is closely related to leaf photosynthesis (von Caemmerer and Farquhar, 1981; Allen et al., 2011; McAusland et al., 2016). During photosynthesis, leaves inevitably lose water to fix carbon gained when they open their stomata (Farooq et al., 2019). The dynamic changes between carbon flux driven by photosynthesis and water flux dominated by transpiration can be characterized by water-use efficiency (WUE; see **Table 1** for the list of abbreviations), also known as intrinsic water use efficiency (WUE_i_) or instantaneous water use efficiency (WUE_inst_) at the leaf level (von Caemmerer and Farquhar, 1981; Fracasso et al., 2017). WUE_i_ is closely correlated with leaf photosynthetic capacity and stomatal behavior (von Caemmerer and Farquhar, 1981). It is commonly used to explain photosynthetic characteristics that are independent of evaporative demand (Ye et al., 2020). WUE_inst_ represents plant dry yield per unit of water loss, which can be considered as the WUE of the whole plant. WUE changes immediately within minutes of becoming water deprived. WUE is regarded as a key physiological indicator for assessing crop growth in arid regions, illuminating the link between crop production and water consumption (McAusland et al., 2016; Fracasso et al., 2017; Bykova et al., 2019; Hatfield and Dold, 2019). Currently, research has been undertaken to explore how crops respond to environmental stress based on the functional characterization of WUE (Gago et al., 2014; Xue et al., 2016; Silva et al., 2020), revealing that an elevated CO_2_ concentration can enhance WUE_inst_ (Wall et al., 2001; Hatfield and Dold, 2019).

**Table 1:** Definitions of the abbreviations.

Abbreviation	Definition	Units
WUE	Water use efficiency	
NRH	Non-rectangular hyperbolic	
WUE_i_	Intrinsic water use efficiency	μmol mol^–1^
WUE_i-max_	Maximum intrinsic water use efficiency	μmol mol^–1^
WUE_inst_	Instantaneous water use efficiency	μmol mmol^–1^
WUE_inst-max_	Maximum instantaneous water use efficiency	μmol mmol^–1^
*A*_n_	Net photosynthetic rate	μmol m^–2^ s^–1^
*A*_nmax_	Maximum net photosynthetic rate	μmol m^–2^ s^–1^
*g*_s_	Stomatal conductance	mol m^–2^ s^–1^
*g*_s-max_	Maximum stomatal conductance	mol m^–2^ s^–1^
*T*_r_	Transpiration rate	mmol m^–2^ s^–1^
*T*_r-max_	Maximum transpiration rate	mmol m^–2^ s^–1^
*I*	Light intensity	μmol m^–2^ s^–1^
*I*_c_	Light compensation point	μmol m^–2^ s^–1^
*I*_sat_	Saturation light intensity corresponding to *A*_nmax_	μmol m^–2^ s^–1^
*I*_g-sat_	Saturation light intensity corresponding to *g*_s-max_	μmol m^–2^ s^–1^
*I*_T-sat_	Saturation light intensity corresponding to *T*_r-max_	μmol m^–2^ s^–1^
*I*_i-sat_	Saturation light intensity corresponding to WUE_i-max_	μmol m^–2^ s^–1^
*I*_inst-sat_	Saturation light intensity corresponding to WUE_inst-max_	μmol m^–2^ s^–1^
*α*	Initial slope of the *A*_n_ *-I* response curve	μmol μmol^–1^
*α*_s_	Initial slope of the *g*_s_ *-I* response curve	mol μmol^–1^
*α*_T_	Initial slope of the *T*_r_ *-I* response curve	mmol μmol^–1^
*α*_i_	Initial slope of the WUE_i_ *-I* response curve	m^2^ s mol^–1^
*α*_inst_	Initial slope of the WUE_inst_ *-I* response curve	m^2^ s mmol^–1^
*R*_d_	Mitochondrial CO_2_ release in the dark	μmol m^–2^ s^–1^
*g*_s0_	Residual stomatal conductance	mol m^–2^ s^–1^
*T*_r0_	Residual transpiration rate	mmol m^–2^ s^–1^
*K*_i_	Residual intrinsic water-use efficiency	μmol mol^–1^
*K*_inst_	Residual instantaneous water use efficiency	μmol mmol^–1^
VPD	Vapor pressure deficit	Kpa
*C*_i_	Intercellular CO_2_ concentration	μmol mol^–1^

However, the process of photosynthesis in plants is inherently dynamic in natural conditions, with the photosynthetic rate exhibiting rapid responses to diverse environmental factors. The intensity of light incident on a leaf experiences fluctuations due to the movement of the sun, clouds, canopies, or the leaf itself, thereby influencing the photosynthetic rate (Gyimah and Nakao, 2007; McAusland et al., 2016). Plants demonstrate varied capabilities in harnessing light of different strengths, ranging from weak to strong, each with specific light requirements (Yan et al., 2019). Characterizing light response of plant photosynthesis is thus essential for elucidating photosynthetic dynamics, cultivation management, and growth potential in changing environments (von Caemmerer and Farquhar, 1981; Gyimah and Nakao, 2007; Yan et al., 2019; Ye et al., 2020). While studies have explored changes in crop WUE (mainly WUE_inst_) under water deficit stress and climate change (Wall et al., 2001; Allen et al., 2011; Fracasso et al., 2017; Hatfield and Dold, 2019; Asadi and Eshghizadeh, 2021), limited information exists on how WUE_i_ and WUE_inst_ respond to variations in light intensity and their evolution under concurrent rising CO_2_ concentration and warming (Hatfield and Dold, 2019).

Accurate characterization of the response curves of net photosynthetic rate (*A*_n_) and WUE to light intensity (*I*) is paramount, serving as a prerequisite for determining key photosynthetic parameters including the maximum net photosynthetic rate (*A*_nmax_), the maximum intrinsic water use efficiency (WUE_i-max_), the maximum instantaneous water use efficiency (WUE_inst-max_), and their corresponding saturation light intensity (i.e., *I*_sat_, *I*_i-sat_, *I*_inst-sat_) (von Caemmerer, 2013; Ye et al., 2020; Yin et al., 2021). This process is essential for evaluating the impact of temperature and CO_2_ concentration on plant WUE and elucidating the relationship between carbon assimilation and water use. Various models have been established to characterize the light response curve of photosynthesis (*A*_n_-*I* curve) in plants, such as the rectangular hyperbola (Thornley, 1998), non-rectangular hyperbolic model (hereafter called NRH model) (Thornley, 1976; Farquhar and Wong, 1984), exponential-based functions (Leakey et al., 2006), and the mechanistic model by Ye et al. (2013) (hereafter called Ye's models). While utilized to predict photosynthesis, most lack assessment of the light response of WUE_i_ and WUE_inst_. Consequently, the NRH model has also been employed to describe WUE_i_-*I* and WUE_inst_-*I* curves. However, studies fitting the *A*_n_-*I* curve have shown the NRH model significantly over/underestimates *A*_nmax_ and cannot estimates *I*_sat_. Recently, we have developed WUE_i_-*I* and WUE_inst_-*I* models based on a mechanistic photosynthesis model, demonstrating accurate fitting of WUE_i_-*I* and WUE_i_-*I* curves for *Glycine max* and *Amaranthus hypochondriacus* at ambient temperature (Ye et al., 2020). However, their applicability across species and environments remains unknown.

In this study, we examined sweet sorghum leaf gas exchange across light levels under varying temperatures and CO_2_ concentrations using a portable LI-6800 photosynthesis system. Light response curves of *A*_n_, stomatal conductance (*g*_s_), transpiration rate (*T*_r_), WUE_i_, and WUE_inst_ were modeled and analyzed. Specifically, we compared the widely used NRH model against the *A*_n_-*I*, *g*_s_-*I*, *T*_r_-*I*, WUE_i_-*I*, and WUE_inst_-*I* models by Ye et al. (Ye and Yu, 2008; Ye et al., 2013; Ye et al., 2020) for accuracy in representing response curves and deriving key photosynthetic parameters. The primary objectives were threefold: (a) to assess the flexibility of the Ye's models and the NRH model in photosynthesis in sweet sorghum under different CO_2_ and temperature conditions; (b) to evaluate applicability of the WUE_i_-*I* and WUE_inst_-*I* models based on the Ye's models and the NRH model to the C_4_ cereal crop sweet sorghum; (c) to elucidate light response characteristics of photosynthesis and WUE in sweet sorghum leaves under short-term exposure to elevated CO_2_ and temperature. These founding not only provide methodological insights for evaluating photosynthesis and water use efficiency dynamics in other crops but also contribute to a deeper understanding of the intricate associations among temperature, CO_2_ and light in modulating sweet sorghum photosynthesis to guide optimal cultivation in a shifting climate.

## Materials and methods

### Plant material and growth conditions

Sweet sorghum cultivar KF-JT-4 were obtained by the Institute of Modern Physics, Chinese Academic of Sciences. This early maturing cultivar was developed via heavy ion irradiation of the parental line KFJT-CK. Thirty seeds were surface sterilized by soaking in 0.025% carbendazim and germinated in Petri dishes on filter paper at 25°C in darkness until radicle emergence. Each seedling was then transplanted into a 1.86 L volume inverted truncated cone-shaped pots (top diameter 15 cm, bottom diameter 10 cm, height 15 cm) filled with field soil. Plants were grown in a controlled environment growth chamber (120 cm long, 75 cm wide, 200 cm high) (RDN-1000E-4, Ningbo Dongnan Instrument Co. Ltd., China) under conditions of 25°C, approximately 260 μmol photons m^−2^ s^−1^ radiation with a 12 h photoperiod, and 70% relative humidity. Plants were routinely watered and fertilized to prevent growth limitation.

After 40 days of cultivation (pre-jointing period), the plants were moved from the growth chamber to a natural laboratory environment to conduct measurements of leaf CO_2_ gas exchange. Subsequent to the completion of each measurement, the plants were promptly returned to the growth chamber. The experiment involved the selection of five healthy and uniform seedlings for analysis. Growth performance of plants is shown in **Supplementary Figure 1**. Key growth traits including plant height, stem diameter, and leaf dimensions were measured with a LI-3000C leaf area meter and are provided in **Supplementary Table S1**.

### Gas exchange measurements and experimental setup

Leaf gas exchange measurement was performed using a portable photosynthesis system (LI-6800, Li-Cor Inc., USA) equipped with an integrated fluorometer chamber head (Li-6800-01A, Li-Cor Inc., USA). From the central leaf, the downward second mature and fully expanded leaf was used for measurement. Prior to each measurement, leaf was clamped in the leaf chamber and acclimated at an irradiance intensity of 1800 μmol m^−2^ s^−1^ for 30~40 min until a steady-state CO_2_ exchange was obtained. The system, featuring red and blue light sources, along with an integrated CO_2_ mixer set to 500 μmol s^−1^ flow rate, facilitated the light-response measurements of CO_2_ gas exchange.

Light-response measurements were systematically conducted in a descending order of light intensity levels: 2000, 1800, 1600, 1400, 1200, 1000, 800, 600, 400, 200, 150, 100, 50, 25, and 0 μmol m^−2^ s^−1^ at the leaf surface level. A minimum wait time of 2 minutes and a maximum of 3 minutes were set at each intensity level before recording data. The instrument automatically aligned reference and sample chamber conditions to ensure accuracy. Each measurement was replicated three times to enhance data reliability and mitigate potential variations.

The internal settings of the LI-6800 photosynthesis system enabled control of leaf temperature and CO_2_ concentration during gas exchange measurements. This allowed simulation of different temperature and CO_2_ treatments to assess short-term photosynthetic responses. Three leaf temperature treatments were imposed: 25, 30, and 35°C at 60-70% relative humidity. CO_2_ treatments included sub-ambient (250 μmol mol^–1^), ambient (410 μmol mol^–1^), and elevated (550 μmol mol^–1^) levels. Under each condition, total leaf exposure spanned 65-80 minutes, encompassing light acclimation (30-40 min) and light response measurements from 0 to 2000 μmol m^−2^ s^−1^ (30-45 min). Measurements occurred between 9:00-11:30 and 14:00-17:00 in April within a ~60 m^2^ laboratory under natural air circulation. This experimental design examined dynamic changes in sweet sorghum photosynthesis under short-term warming and elevated CO_2_ to elucidate plant responses to varying environmental conditions.

### Analytical models and calculation

The dependence of *A*_n_ on *I* in Ye's models (Ye et al., 2013) is expressed as follows:


(1) $$A_{\text{n}}=\alpha \frac{1-\beta I}{1+\gamma I}I-R_{\text{d }}$$

where *A*_n_ is the net photosynthetic rate, *α* is the initial slope of the *A*_n_ *-I* response curve, *β* and *γ* are the two parameters reflecting light limitation and light saturation, respectively, and *R*_d_ is the dark respiration rate.

The saturation light intensity (*I*_sat_) corresponding to *A*_nmax_ can be calculated by the following equations:


(2) $$I_{\text{sat}}=\frac{\sqrt{\left(\beta +\gamma \right)/\beta }-1}{\gamma }$$

and


(3) $$A_{\text{nmax}}=\alpha {\left(\frac{\sqrt{\beta +\gamma }-\sqrt{\beta }}{\gamma }\right)}^{2}-R_{\text{d}}$$

The *g*_s_-*I* model established by Ye and Yu (2008) was given as follows:


(4) $$g_{\text{s}}=\alpha_{\text{s}}\frac{1-\beta_{\text{s}}I}{1+\gamma_{\text{s}}I}I+g_{\text{s}0}$$

where *α*_s_ is the initial slope of the *g*_s_ *-I* response curve, *β*_s_ and *γ*_s_ are the two coefficients independent of *I*. *g*_s0_ is the residual stomatal conductance when *I* = 0.

The saturation light intensity (*I*_g-sat_) corresponding to the maximum stomatal conductance (*g*_s-max_) can be found by the following equations:


(5) $$I_{\text{g-sat}}=\frac{\sqrt{\left(\beta_{\text{s}}+\gamma_{\text{s}}\right)/\beta_{\text{s}}}-1}{\gamma_{\text{s}}}$$

and


(6) $$g_{\text{s-max}}=\alpha_{\text{s}}{\left(\frac{\sqrt{\beta_{\text{s}}+\gamma_{\text{s}}}-\sqrt{\beta_{\text{s}}}}{\gamma_{\text{s}}}\right)}^{2}+g_{\text{s}0}$$

WUE_i_ can be calculated by the ratio of *A*_n_ to *g*_s_ (*A*_n_/*g*_s_) in μmol mol^−1^. The WUE_i_-*I* model established by Ye et al. (2020) was given as follows:


(7) $${\text{WUE}}_{\text{i}}=\alpha_{\text{i}}\frac{1-\beta_{\text{i}}I}{1+\gamma_{\text{i}}I}I-K_{\text{i}}$$

where *α*_i_ denotes the initial slope of WUE_i_ *-I* response curve, *β*_i_ and *γ*_i_ are the two coefficients that are independent of *I*, and *K*_i_ is the residual WUE_i_.

The saturated light intensity (*I*_i-sat_) corresponding to the maximum WUE_i_ (WUE_i-max_) can be calculated by the following equations:


(8) $$I_{\text{i-sat}}=\frac{\sqrt{\left(\beta_{\text{i}}+\gamma_{\text{i}}\right)/\gamma_{\text{i}}}-1}{\gamma_{\text{i}}}$$

and


(9) $${\text{WUE}}_{\text{i-max}}=\alpha_{\text{i}}{\left(\frac{\sqrt{\beta_{\text{i}}+\gamma_{\text{i}}}-\sqrt{\beta_{\text{i}}}}{\gamma_{\text{i}}}\right)}^{2}-K_{\text{i}}$$

WUE_inst_ is expressed as the ratio between *A*_n_ and *T*_r_ (*A*_n_/*T*_r_) in μmol mmol^−1^. The WUE_inst_-*I* model established by Ye et al. (2020) was given as follows:


(10) $${\text{WUE}}_{\text{inst}}=\alpha_{\text{inst}}\frac{1-\beta_{\text{inst}}I}{1+\gamma_{\text{inst}}I}I-K_{\text{inst}}$$

where *α*_inst_ denotes the initial slope of WUE_inst_ *-I* response curve, *β*_inst_ and *γ*_inst_ are the two coefficients that are independent of *I*, and *K*_inst_ is the residual WUE_inst_.

The saturation light intensity (*I*_inst-sat_) corresponding to the maximum WUE_inst_ (WUE_inst-max_) can be calculated by the following equations:


(11) $$I_{\text{inst-sat}}=\frac{\sqrt{\left(\beta_{\text{inst}}+\gamma_{\text{inst}}\right)/\gamma_{\text{inst}}}-1}{\gamma_{\text{inst}}}$$

and


(12) $${\text{WUE}}_{\text{inst-max}}=\alpha_{\text{inst}}{\left(\frac{\sqrt{\beta_{\text{inst}}+\gamma_{\text{inst}}}-\sqrt{\beta_{\text{inst}}}}{\gamma_{\text{inst}}}\right)}^{2}-K_{\text{inst}}$$

The dependence of *A*_n_ on *I* in NRH model (Thornley, 1976; Farquhar and Wong, 1984) is expressed as follows:


(13) $$A_{\text{n}}=\frac{\alpha I+A_{\text{nmax}}-\sqrt{{\left(\alpha I+A_{\text{nmax}}\right)}^{2}-4\alpha \theta IA_{\text{nmax}}}}{2\theta }-R_{\text{d}}$$

where *A*_n_ is the net photosynthetic rate at light intensity *I*, *A*_nmax_ is the maximum net photosynthetic rate, *α* is the initial slope of *A*_n_ *-I* response curve, *θ* is the convexity of curve, and *R*_d_ is the dark respiration rate. The NRH model has also been used to characterize the responses of *g*_s_, *T*_r_, WUE_i_, and WUE_inst_ to *I* (Ye et al., 2020).

### Statistical analysis

The data of *A*_n_-*I*, *g*_s_ *-I*, *T*_r_ *-I*, WUE_i_-*I*, and WUE_int_-*I* were fitted by non-linear regression using the Levenberg-Marquardt algorithm in SPSS version 24.0 and the *Photosynthesis Model Simulation Software* (PMSS, http://photosynthetic.sinaapp.com). Goodness of fit was evaluated by the coefficient of determination (*R* ^2^). Student’s *t*-tests assessed differences in parameter values between the photosynthetic models and between fitted and observed values. The effects of temperature and CO_2_ on photosynthetic parameters were analyzed by one-way ANOVA with Duncan’s *post-hoc* test. Data were expressed as group mean ± standard errors (*n* = 3). Statistical significance was accepted at *p*<0.05.

## Results

### Performance of models in reproducing light response curves and quantifying photosynthetic traits

**Figure 1:**
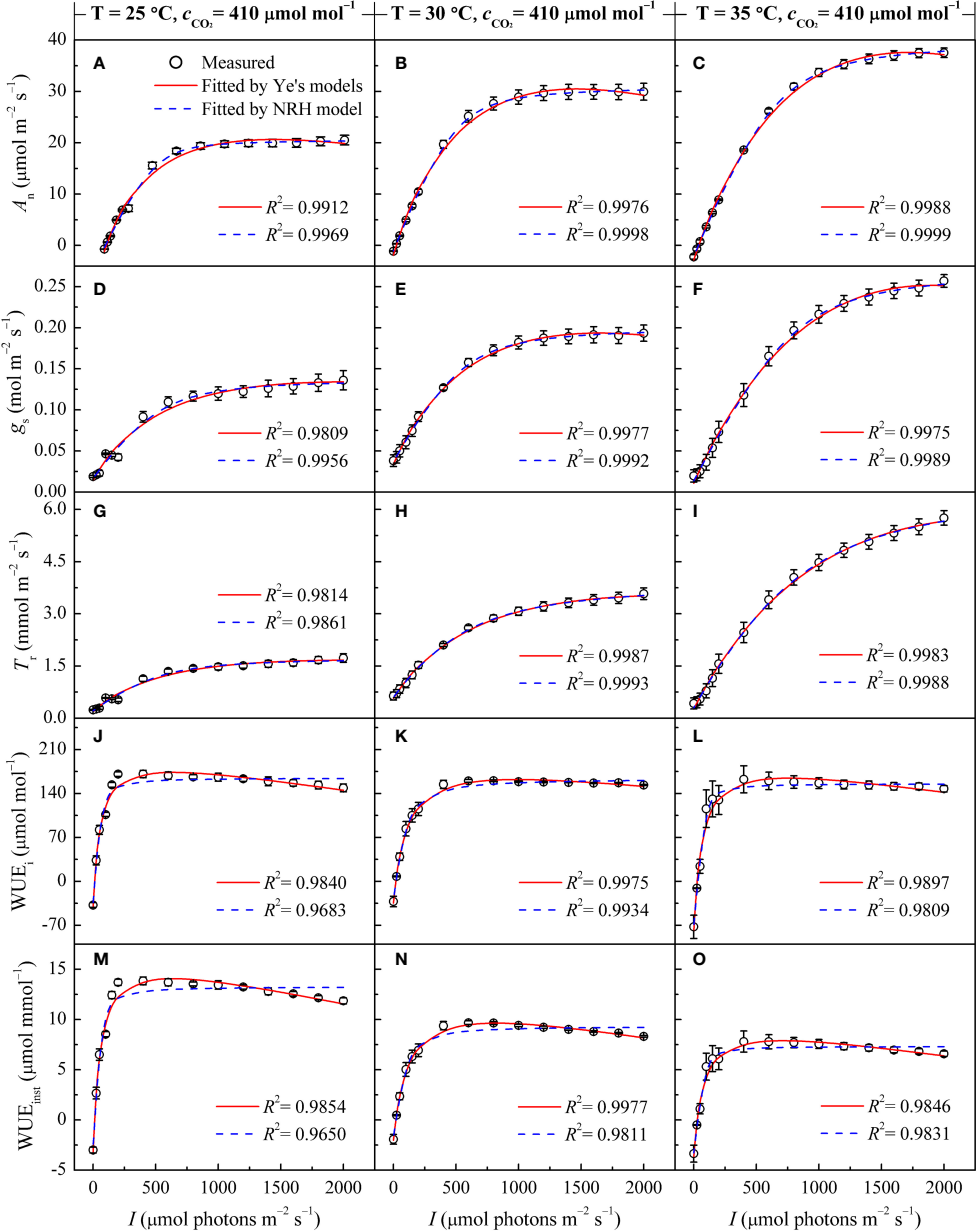
Changes in gas exchange parameters of sweet sorghum leaves across varied air temperatures (25, 30, and 35°C) under atmospheric CO_2_ level (410 μmol mol^−1^), alongside comparison of light response curves fitted by the Ye's models and the NRH model. **(A-C)** present the net photosynthetic rate (*A*_n_); **(D-F)** stomatal conductance (*g*_s_); **(G-I)** Transpiration rate (*T*_r_); **(J-L)** intrinsic water-use efficiency (WUE_i_); and **(M-O)** instantaneous water-use efficiency (WUE_inst_).

Both the Ye's models (Equations 1, 4) and the NRH model (Equation 13) successfully reproduced the *A*_n_-*I*, *g*_s_-*I*, and *T*_r_-*I* curves of sweet sorghum under varying temperatures (**Figures 1A-I**). The NRH model showed slightly higher *R* ^2^ values in comparison to the Ye's models. However, NRH model predictions for *A*_nmax_ values significantly exceeded observed values (**Supplementary Table 2**) (*p*<0.05). Additionally, *g*_s-max_ and *T*_r-max_ values exhibited disparities with the measured values, either overestimated or underestimated. Focusing on the curves depicting the relationship between WUE_i_ or WUE_inst_ and *I*, the Ye's WUE models (Equations 7, 10) outperformed the NRH model across all temperature treatments by accurately captured the decline in WUE_i_ and WUE_inst_ as *I* increased under high light intensity, a trend not reflected by the NRH model (**Figures 1J-O**). WUE_i-max_ and WUE_inst-max_ values from the Ye's WUE models closely matched observed values, with no statistically significant differences (*p*>0.05) (**Supplementary Table 2**). In addition, saturation light intensities (*I*_sat_, *I*_g-sat_, *I*_T-sat_, *I*_i-sat_ and *I*_inst-sat_) corresponding to *A*_nmax_, *g*_s-max_, *T*_r-max_, WUE_i-max_ and WUE_inst-max_ were directly obtained from the Ye's models fitting but not from the NRH model fitting (**Table 2**). WUE_i-max_ and WUE_inst-max_ values from the NRH model were significantly overestimated compared to observed values across all temperature conditions (*p*<0.05) (**Supplementary Table 2**).

**Table 2:** Comparison analysis of photosynthetic parameters in sweet sorghum under varied temperature conditions at 410 μmol mol^−1^ CO_2_ based on fitting values from light response curves using different photosynthetic models.

Photosynthetic parameters	Fitted by the Ye's models			Fitted by the NRH model		
	25 °C	30 °C	35 °C	25 °C	30 °C	35 °C
*α* (μmol μmol^–1^)	0.0726 ± 0.0048 ^b^	0.0839 ± 0.0035 ^a^	0.0732 ± 0.0026 ^b^	0.0459 ± 0.0039 ^b^	0.0598 ± 0.0025 ^a^	0.0575 ± 0.0013 ^a^
*A*_nmax_ (μmol m^–2^ s^–1^)	20.70 ± 0.73 ^c^	30.54 ± 0.76 ^b^	37.67 ± 0.96 ^a^	21.07 ± 1.02 ^c^	32.25 ± 1.68 ^b^	42.30 ± 1.21 ^a^
*I*_sat_ (μmol m^–2^ s^–1^)	1434.87 ± 92.87 ^b^	1460.77 ± 169.70 ^b^	1710.8 ± 31.3 ^a^	―	―	―
*I*_c_ (μmol m^–2^ s^–1^)	16.90 ± 0.11 ^b^	23.30 ± 1.16 ^b^	39.27 ± 5.19 ^a^	8.08 ± 2.03 ^b^	18.01 ± 0.91 ^b^	36.87 ± 6.02 ^a^
*R*_d_ (μmol m^–2^ s^–1^)	1.19 ± 0.08 ^b^	1.89 ± 0.02 ^b^	2.80 ± 0.44 ^a^	0.39 ± 0.12 ^b^	1.07 ± 0.05 ^b^	2.12 ± 0.39 ^a^
*α*_s_ (mol μmol^–1^)	0.0003 ± 0.0000 ^b^	0.0004 ± 0.0000 ^a^	0.0004 ± 0.0000 ^a^	0.0002 ± 0.0000 ^b^	0.0003 ± 0.0000 ^a^	0.0003 ± 0.0000 ^a^
*g*_s-max_ (mol m^–2^ s^–1^)	0.138 ± 0.012 ^c^	0.194 ± 0.010 ^b^	0.252 ± 0.008 ^a^	0.123 ± 0.012 ^c^	0.167 ± 0.011 ^b^	0.260 ± 0.004 ^a^
*I*_g-sat_ (μmol m^–2^ s^–1^)	1765.96 ± 158.02 ^a^	1654.96 ± 22.29 ^a^	1900.89 ± 74.45 ^a^	―	―	―
*g*_s0_ (mol m^–2^ s^–1^)	0.014 ± 0.001 ^ab^	0.033 ± 0.007 ^a^	0.011 ± 0.008 ^b^	0.017 ± 0.001 ^ab^	0.036 ± 0.007 ^a^	0.013 ± 0.008 ^b^
*α*_r_ (mmol μmol^–1^)	0.0034 ± 0.0002 ^b^	0.0060 ± 0.0006 ^a^	0.0072 ± 0.0006 ^a^	0.0025 ± 0.0002 ^b^	0.0068 ± 0.0005 ^a^	0.0065 ± 0.0006 ^a^
*T*_r-max_ (mmol m^–2^ s^–1^)	1.68 ± 0.11 ^c^	3.56 ± 0.15 ^b^	5.83 ± 0.27 ^a^	1.54 ± 0.11 ^c^	4.01 ± 0.48 ^b^	6.52 ± 0.26 ^a^
*I*_T-sat_ (μmol m^–2^ s^–1^)	2292.28 ± 392.15 ^a^	2578.42 ± 319.24 ^a^	2663.16 ± 310.52 ^a^	―	―	―
*T*_r0_ (mmol m^–2^ s^–1^)	0.18 ± 0.02 ^a^	0.56 ± 0.12 ^a^	0.25 ± 0.16 ^a^	0.21 ± 0.02 ^a^	0.53 ± 0.11 ^a^	0.28 ± 0.16 ^a^
*α*_i_ (m^2^ s mol^–1^)	4.7330 ± 0.3587 ^a^	2.3849 ± 0.6450 ^b^	3.9871 ± 1.412 ^ab^	2.8690 ± 0.5946 ^a^	1.5512 ± 0.5053 ^a^	2.7595 ± 0.6064 ^a^
WUE_i-max_ (μmol mol^–1^)	174.17 ± 3.62 ^a^	163.14 ± 2.14 ^a^	151.34 ± 6.46 ^b^	200.22 ± 9.84 ^a^	192.49 ± 8.96 ^a^	225.74 ± 18.60 ^a^
*I*_i-sat_ (μmol m^–2^ s^–1^)	615.35 ± 32.16 ^a^	965.35 ± 76.79 ^a^	859.94 ± 222.47 ^a^	―	―	―
*K*_i_ (μmol mol^–1^)	40.74 ± 1.79 ^b^	34.91 ± 8.32 ^b^	75.18 ± 19.12 ^a^	35.30 ± 4.22 ^b^	29.40 ± 9.64 ^b^	72.47 ± 17.54 ^a^
*α*_inst_ (m^2^ s mmol^–1^)	0.3665 ± 0.0274 ^a^	0.1367 ± 0.0354 ^b^	0.1706 ± 0.0618 ^b^	0.2204 ± 0.0424 ^a^	0.0850 ± 0.0281 ^b^	0.1209 ± 0.029 ^ab^
WUE_inst-max_ (μmol mmol^–1^)	14.12 ± 0.22 ^a^	9.70 ± 0.20 ^b^	7.21 ± 0.35 ^c^	16.03 ± 0.64 ^a^	10.94 ± 0.58 ^b^	10.45 ± 0.89 ^b^
*I*_inst-sat_ (μmol m^–2^ s^–1^)	602.71 ± 18.01 ^a^	799.13 ± 63.61 ^a^	728.23 ± 30.09 ^a^	―	―	―
*K*_inst_ (μmol mmol^–1^)	3.22 ± 0.13 ^a^	2.07 ± 0.48 ^a^	3.43 ± 0.88 ^a^	2.75 ± 0.33 ^a^	1.66 ± 0.60 ^a^	3.32 ± 0.80 ^a^

Mean and standard errors of three replicates are shown. Statistically significant differences (*p*<0.05) between temperature treatment groups are denoted by different letters under the Ye's models and the NRH model.

**Figure 2:**
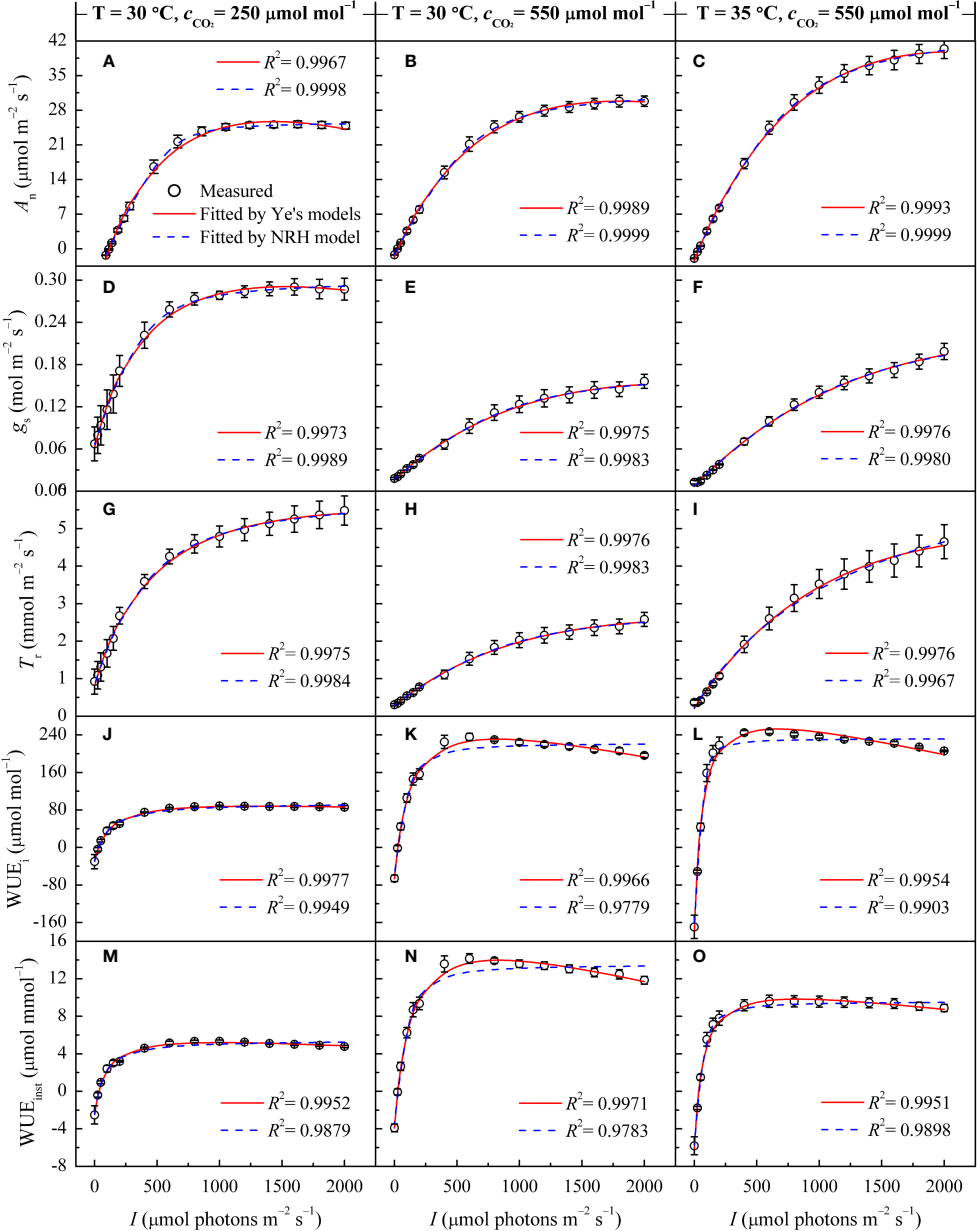
Changes in gas exchange parameters of sweet sorghum leaves across varied CO_2_ concentrations, as well as comparison of light response curves fitted by the Ye's models and the NRH model. Figures **(A, B)**, **(D, E)**, **(G, H)**, **(J, K)** and **(M, N)** present changes in *A*_n_, *g*_s_, *T*_r_, WUE_i_, and WUE_inst_ at 30°C under CO_2_ concentrations of 250 and 550 μmol mol^−1^, respectively. Figures **(C, F, I, L)** and **(O)** show the changes in *A*_n_, *g*_s_, *T*_r_, WUE_i_, and WUE_inst_ at 35°C under a CO_2_ concentrations of 550 μmol mol^−1^.

Similar to its performance under varying temperatures, the NRH model was employed to fit the *A*_n_-*I* curves under different CO_2_ concentrations, yielding *A*_nmax_ values that exceeded their observed counterparts (**Supplementary Table 3**). While successfully reproducing the *g*_s_-*I* and *T*_r_-*I* curves (**Figures 2D-I**), the NRH model struggled to accurately predict *g*_s-max_ and *T*_r-max_, consequently failing to determine corresponding saturation light intensities (**Table 3**). In contrast, the Ye's models performed exceptionally well in fitting these curves. When used to fit the WUE_i_-*I* and WUE_inst_-*I* curves under different CO_2_ concentrations, Ye's WUE models consistently demonstrated outstanding performance, with *R* ^2^ exceeding 0.995 (**Figures 2J-O**). The fitted WUE_i-max_ and WUE_inst-max_ values showed no significant differences from their respective measured values (*p*>0.05) (**Supplementary Table 3**). Nevertheless, the NRH model inadequately captured changes in the responses of WUE_i_ and WUE_inst_ to *I*, particularly evident in a low fitting coefficient for WUE_inst_-*I* (**Figures 2J-O**). Consequently, the fitted WUE_i-max_ and WUE_inst-max_ values exceeded their measured counterparts by more than 20% (**Supplementary Table 3**).

**Table 3:** Comparison analysis of photosynthetic parameters in sweet sorghum under varied CO_2_ levels at 30°C based on fitting values from light response curves using different photosynthetic models.

Photosynthetic parameters	Fitted by the Ye's models			Fitted by the NRH model		
	250 μmol mol^–1^	410 μmol mol^–1^	550 μmol mol^–1^	250 μmol mol^–1^	410 μmol mol^–1^	550 μmol mol^–1^
*α* (μmol μmol^–1^)	0.0728 ± 0.0078 ^ab^	0.0839 ± 0.0035 ^a^	0.0610 ± 0.0068 ^b^	0.0491 ± 0.0037 ^a^	0.0598 ± 0.0025 ^a^	0.0481 ± 0.0048 ^a^
*A*_nmax_ (μmol m^–2^ s^–1^)	25.84 ± 0.65 ^b^	30.54 ± 0.76 ^a^	29.88 ± 1.07 ^a^	27.01 ± 0.71 ^b^	32.25 ± 1.68 ^a^	33.43 ± 1.10 ^a^
*I*_sat_ (μmol m^–2^ s^–1^)	1393.79 ± 71.49 ^b^	1460.8 ± 169.7 ^b^	1782.36 ± 30.13 ^a^	―	―	―
*I*_c_ (μmol m^–2^ s^–1^)	30.42 ± 2.13 ^a^	23.30 ± 1.16 ^b^	28.87 ± 1.90 ^ab^	25.56 ± 2.49 ^a^	18.01 ± 0.91 ^b^	25.00 ± 1.61 ^a^
*R*_d_ (μmol m^–2^ s^–1^)	2.09 ± 0.08 ^a^	1.89 ± 0.02 ^ab^	1.70 ± 0.13 ^b^	1.23 ± 0.03 ^a^	1.07 ± 0.05 ^a^	1.19 ± 0.09 ^a^
*α*_s_ (mol μmol^–1^)	0.0007 ± 0.0000 ^a^	0.0004 ± 0.0000 ^b^	0.0002 ± 0.0000 ^c^	0.0005 ± 0.0000 ^a^	0.0003 ± 0.0000 ^b^	0.0001 ± 0.0000 ^c^
*g*_s-max_ (mol m^–2^ s^–1^)	0.293 ± 0.011 ^a^	0.194 ± 0.010 ^b^	0.156 ± 0.008 ^c^	0.237 ± 0.040 ^a^	0.167 ± 0.011 ^a^	0.161 ± 0.002 ^a^
*I*_g-sat_ (μmol m^–2^ s^–1^)	1484.64 ± 184.44 ^b^	1654.9 ± 22.3 ^b^	2533.57 ± 328.53 ^a^	―	―	―
*g*_s0_ (mol m^–2^ s^–1^)	0.061 ± 0.026 ^a^	0.033 ± 0.007 ^a^	0.016 ± 0.001 ^a^	0.066 ± 0.026 ^a^	0.036 ± 0.007 ^a^	0.017 ± 0.001 ^a^
*α*_r_ (mmol μmol^–1^)	0.0123 ± 0.0007 ^a^	0.0060 ± 0.0006 ^b^	0.0028 ± 0.0003 ^c^	0.0105 ± 0.0005 ^a^	0.0068 ± 0.0005 ^b^	0.0025 ± 0.0002 ^c^
*T*_r-max_ (mmol m^–2^ s^–1^)	5.48 ± 0.36 ^a^	3.56 ± 0.15 ^b^	2.62 ± 0.13 ^c^	5.13 ± 0.76 ^a^	4.01 ± 0.48 ^ab^	2.74 ± 0.04 ^b^
*I*_T-sat_ (μmol m^–2^ s^–1^)	2378.80 ± 142.08 ^a^	2578.4 ± 319.2 ^a^	2837.28 ± 396.25 ^a^	―	―	―
*T*_r0_ (mmol m^–2^ s^–1^)	0.79 ± 0.38 ^a^	0.56 ± 0.12 ^a^	0.27 ± 0.02 ^b^	0.84 ± 0.37 ^a^	0.53 ± 0.11 ^a^	0.29 ± 0.02 ^b^
*α*_i_ (m^2^ s mol^–1^)	1.4960 ± 0.8689 ^a^	2.3849 ± 0.6450 ^a^	3.1903 ± 0.5037 ^a^	1.1939 ± 0.7342 ^a^	1.5512 ± 0.5053 ^a^	2.0450 ± 0.2548 ^a^
WUE_i-max_ (μmol mol^–1^)	88.60 ± 2.71 ^c^	163.14 ± 2.14 ^b^	232.37 ± 6.57 ^a^	120.79 ± 13.65 ^c^	192.49 ± 8.96 ^b^	281.68 ± 12.86 ^a^
*I*_i-sat_ (μmol m^–2^ s^–1^)	1227.71 ± 62.57 ^a^	965.35 ± 76.79 ^b^	806.83 ± 54.02 ^b^	―	―	―
*K*_i_ (μmol mol^–1^)	30.38 ± 15.56 ^b^	34.91 ± 8.32 ^b^	66.68 ± 7.90 ^a^	28.68 ± 16.28 ^b^	29.40 ± 9.64 ^b^	57.72 ± 7.35 ^a^
*α*_inst_ (m^2^ s mmol^–1^)	0.1307 ± 0.0583 ^a^	0.1367 ± 0.0354 ^a^	0.1849 ± 0.0293 ^a^	0.1009 ± 0.0464 ^a^	0.0850 ± 0.0281 ^a^	0.1196 ± 0.0159 ^a^
WUE_inst-max_ (μmol mmol^–1^)	5.26 ± 0.19 ^c^	9.70 ± 0.20 ^b^	14.05 ± 0.47 ^a^	7.65 ± 0.94 ^c^	10.94 ± 0.58 ^b^	16.95 ± 0.91 ^a^
*I*_inst-sat_ (μmol m^–2^ s^–1^)	985.69 ± 14.90 ^a^	799.13 ± 63.61 ^b^	815.71 ± 49.94 ^b^	―	―	―
*K*_inst_ (μmol mmol^–1^)	2.53 ± 0.98 ^a^	2.07 ± 0.48 ^a^	3.91 ± 0.47 ^a^	2.39 ± 1.05 ^a^	1.66 ± 0.60 ^a^	3.38 ± 0.45 ^a^

Mean and standard errors of three replicates are shown. Statistically significant differences (*p*<0.05) between temperature treatment groups are denoted by different letters under the Ye's models and the NRH model.

### Light response curves of *A*_n_, *g*_s_, *T*_r_, WUE_i_ and WUE_inst_ in sweet sorghum under varied leaf temperatures

In **Figures 1A-I**, the responses of *A*_n_, *g*_s_, and *T*_r_ to varying *I* under different leaf temperatures exhibited comparable patterns. Below 800 μmol m^−2^ s^−1^, *A*_n_, *g*_s_, and *T*_r_ increased rapidly with increasing *I* until reaching their respective peaks (*A*_nmax_, *g*_s-max_, and *T*_r-max_) at the specific saturation light intensities (*I*_sat_, *I*_g-sat_, and *I*_T-sat_). Notably, at high light intensity levels (*I >*1400 μmol m^−2^ s^−1^), a decrease in *A*_n_ with increasing *I* was not observed, indicating the absence of photoinhibition. **Figures 1J-O** illustrates the variations in WUE_i_ and WUE_inst_ in response to *I* at different temperatures. Both WUE_i_ and WUE_inst_ showed almost linear increases within the 200 μmol m^−2^ s^−1^ range, indicating improving WUE with increasing *I*. In other words, the plant became more efficient in utilizing water for photosynthesis relative to the amount of light it received. However, upon reaching the WUE_i-max_ and WUE_inst-max_ at corresponding *I*_i-sat_ and *I*_inst-sat_, gradual decreases were observed (**Table 2**), suggesting that additional light did not proportionally increase photosynthesis but potentially led to increased water loss without a corresponding increase in carbon gain. It should be noted that *T*_r_ for sweet sorghum under varying CO_2_ and temperature conditions did not reach saturation within the values of *I* applied for the *T*_r_-*I* curve and hence values for *I*_T-sat_ (and *T*_r-max_) are an extrapolation.

The photosynthetic processes in sweet sorghum leaves were significantly influenced by temperature (one-way and two-way ANOVA *p*<0.05) (**Tables 2**, **4**). At an ambient CO_2_ level (410 μmol mol^−1^), elevating temperature led to significant increases in *A*_nmax_, *g*_s-max_, and *T*_r-max_ (*p*<0.05). This rise in temperature also corresponded with amplified values in *I*_sat_, *I*_c_, and *R*_d_. However, both WUE_i-max_ and WUE_inst-max_ consistently declined with increasing temperature, with WUE_inst-max_ displaying the most pronounced decrease and significant differences across temperature levels (*p*<0.05). For instance, increasing the temperature from 25°C to 30°C caused increase of 47.54%, 40.58%, and 111.91% in *A*_nmax_, *g*_s-max_, and *T*_r-max_, respectively (*p*<0.05). No significant changes were observed in *I*_sat_ and *I*_g-sat_, while *I*_T-sat_ exhibited a non-significant increase (*p*>0.05). WUE_i-max_ only decreased by 6.33% without significant differences, whereas WUE_inst-max_ significantly decreased by 31.30% (**Table 2**). Further increasing the temperature from 30°C to 35°C, despite the moderated enhancements in *A*_nmax_, *g*_s-max_, and *T*_r-max_ (increases of 23.34%, 29.90%, and 63.76%, respectively), the reduction in WUE_i-max_ expanded to 7.23%, and WUE_inst-max_ declined by 25.67%. Higher temperature raised the saturation light intensities corresponding to WUE_i-max_ and WUE_inst-max_ (**Table 2**). However, at an elevated CO_2_ level (550 μmol mol^−1^), rising temperature from 30°C to 35°C significantly increased WUE_i-max_, while expanding the decline of WUE_inst-max_ to 29.47% due to a substantial 88.55% increase in *T*_r-max_ (**Supplementary Table 4**).

**Table 4:** Summary of two-way ANOVA analysis between the effects of temperature and CO_2_ concentration on key photosynthetic parameters of sweet sorghum.

Dependent variables	Factors	DF	F	*p* value
*α* (μmol μmol^–1^)	Temperature	1	1.072	0.321
	CO_2_ concentration	1	22.831	< 0.001
	Temperature × CO_2_ concentration	1	4.981	**0.045**
*A*_nmax_ (μmol m^–2^ s^–1^)	Temperature	1	50.299	< 0.001
	CO_2_ concentration	1	0.406	0.536
	Temperature × CO_2_ concentration	1	1.411	0.258
*I*_sat_ (μmol m^–2^ s^–1^)	Temperature	1	76.254	< 0.001
	CO_2_ concentration	1	122.789	< 0.001
	Temperature × CO_2_ concentration	1	0.285	0.603
*I*_c_ (μmol m^–2^ s^–1^)	Temperature	1	27.790	< 0.001
	CO_2_ concentration	1	1.029	0.331
	Temperature × CO_2_ concentration	1	1.606	0.229
*R*_d_ (μmol m^–2^ s^–1^)	Temperature	1	19.273	0.001
	CO_2_ concentration	1	2.247	0.160
	Temperature × CO_2_ concentration	1	0.232	0.639
*g*_s-max_ (mol m^–2^ s^–1^)	Temperature	1	63.950	< 0.001
	CO_2_ concentration	1	25.771	< 0.001
	Temperature × CO_2_ concentration	1	0.007	0.934
*T*_r-max_ (mmol m^–2^ s^–1^)	Temperature	1	81.140	< 0.001
	CO_2_ concentration	1	12.919	0.004
	Temperature × CO_2_ concentration	1	0.009	0.927
WUE_i-max_ (μmol mol^–1^)	Temperature	1	2.191	0.165
	CO_2_ concentration	1	514.088	< 0.001
	Temperature × CO_2_ concentration	1	20.812	**0.001**
WUE_inst-max_ (μmol mmol^–1^)	Temperature	1	78.809	< 0.001
	CO_2_ concentration	1	89.111	< 0.001
	Temperature × CO_2_ concentration	1	4.911	**0.047**

The bold p value indicates a significant interactive effect between temperature and CO_2_ concentration on the parameter.

**Table 5:** Comparison of results fitted by the Ye's models and the NRH model with measured data in sweet sorghum under elevated CO_2_ concentration (410 μmol mol^−1^) and high temperature (35°C) in combination.

Photosynthetic parameters	Temperature of 35°C and CO_2_ concentration of 550 μmol mol^–1^		
	Fitted by the Ye's models	Fitted by the NRH model	Measured by LI-6800
*α* (μmol μmol^–1^)	0.0649 ± 0.0033 ^a^	0.0557 ± 0.003 ^a^	―
*A*_nmax_ (μmol m^–2^ s^–1^)	39.87 ± 1.92 ^b^	47.62 ± 2.11 ^a^	40.42 ± 1.98 ^b^
*I*_sat_ (μmol m^–2^ s^–1^)	2064.90 ± 45.82 ^a^	―	2000.00 ± 0.00 ^a^
*I*_c_ (μmol m^–2^ s^–1^)	38.64 ± 2.9 ^a^	37.15 ± 2.95 ^a^	37.99 ± 1.25 ^a^
*R*_d_ (μmol m^–2^ s^–1^)	2.43 ± 0.18 ^a^	2.04 ± 0.14 ^ab^	1.95 ± 0.08 ^b^
*α*_s_ (mol μmol^–1^)	0.0002 ± 0.0000 ^a^	0.0002 ± 0.0000 ^a^	―
*g*_s-max_ (mol m^–2^ s^–1^)	0.215 ± 0.01 ^b^	0.256 ± 0.013 ^a^	0.198 ± 0.012 ^b^
*I*_g-sat_ (μmol m^–2^ s^–1^)	3484.01 ± 395.74	―	>2000
*g*_s0_ (mol m^–2^ s^–1^)	0.006 ± 0.003 ^a^	0.007 ± 0.003 ^a^	0.013 ± 0.003 ^a^
*α*_r_ (mmol μmol^–1^)	0.0053 ± 0.0007 ^a^	0.0049 ± 0.0004 ^a^	―
*T*_r-max_ (mmol m^–2^ s^–1^)	4.94 ± 0.45 ^b^	7.03 ± 0.23 ^a^	4.65 ± 0.45 ^b^
*I*_T-sat_ (μmol m^–2^ s^–1^)	2561.44 ± 123.51	―	>2000
*T*_r0_ (mmol m^–2^ s^–1^)	0.21 ± 0.06 ^a^	0.24 ± 0.04 ^a^	0.37 ± 0.07 ^a^
*α*_i_ (m^2^ s mol^–1^)	9.1759 ± 1.8803 ^a^	4.5104 ± 0.7748 ^b^	―
WUE_i-max_ (μmol mol^–1^)	255.46 ± 5.26 ^b^	404.47 ± 27.6 ^a^	247.05 ± 4.14 ^b^
*I*_i-sat_ (μmol m^–2^ s^–1^)	644.44 ± 49.65 ^a^	―	525.00 ± 47.87 ^a^
*K*_i_ (μmol mol^–1^)	181.31 ± 25.48 ^a^	170.53 ± 26.28 ^a^	169.55 ± 24.90 ^a^
*α*_inst_ (m^2^ s mmol^–1^)	0.2941 ± 0.0656 ^a^	0.1633 ± 0.0292 ^a^	―
WUE_inst-max_ (μmol mmol^–1^)	9.91 ± 0.64 ^b^	15.36 ± 1.24 ^a^	9.66 ± 0.64 ^b^
*I*_inst-sat_ (μmol m^–2^ s^–1^)	816.50 ± 54.83 ^a^	―	950.02 ± 132.29 ^a^
*K*_inst_ (μmol mmol^–1^)	6.19 ± 0.99 ^a^	5.75 ± 0.93 ^a^	5.82 ± 0.96 ^a^

Mean and standard errors of three replicates are shown. Different letters denote statistically significant differences (*p*<0.05) among the values fitted by the Ye's models, the values fitted by the NRH model, and the measured values.

### Light response curves of *A*_n_, *g*_s_, *T*_r_, WUE_i_ and WUE_inst_ in sweet sorghum under varied air CO_2_ concentrations

In **Figures 2A-D**, under sub-ambient CO_2_ concentration, *A*_n_ and *g*_s_ exhibited consistent response trends to *I*, rapidly increasing and reaching *A*_n-max_ and *g*_s-max_ at corresponding *I*_sat_ and *I*_g-sat_. With increasing CO_2_ concentration, the responses of *g*_s_ and *T*_r_ to *I* gradually shifted from a single-peaked pattern to an approximately linear increase (**Figures 2D-I**). Under elevated CO_2_, *A*_n_ reached saturation within the measured light intensity range, whereas *g*_s_ and *T*_r_ increased nearly linearly with *I*, especially at 35°C (**Figures 2F, I**). Across varying CO_2_ concentrations, the patterns in WUE_i_-*I* and WUE_inst_-*I* curves displayed distinct divisions into light-limited, light-saturated, and light-inhibited segments (**Figures 2J-O**). At high light intensity (*I*>1000 μmol m^−2^ s^−1^) under elevated CO_2_, both WUE_i_ and WUE_inst_ showed a significant reduction.

At a specific temperature (30°C), increasing CO_2_ concentration from 250 to 410 μmol mol^−1^ resulted in increased *A*_nmax_ and *α* for sweet sorghum, while decreasing *I*_c_ and *R*_d_ (**Table 3**). *I*_sat_ corresponding to *A*_nmax_ remained unchanged. However, further increasing the concentration to 550 μmol mol^−1^ did not cause a continual increase in *A*_nmax_; instead, it raised both *I*_sat_ and *I*_c_. Elevated CO_2_ significantly weakened photosynthesis under light-limited conditions, as evidenced by reductions in *α*, *α*_g_, and *α*_T_ (**Table 3**). For *g*_s_ and *T*_r_, their values decreased as CO_2_ levels rose, and their response curves to light became less steep (**Figures 2D-I**), leading to a significant decrease in *g*_s-max_ and *T*_r-max_ (*p*<0.05). Consequently, there was a substantial increase in both WUE_i-max_ and WUE_inst-max_ with elevated CO_2_ (*p*<0.05). Specifically, upon elevating CO_2_ concentration from 410 to 550 μmol mol^−1^, *g*_s-max_ decreased by 19.59%, *T*_r-max_ by 26.40%, while WUE_i-max_ increased by 42.44% and WUE_inst-max_ by 44.85% (**Table 5**).

At a high temperature of 35°C, elevated CO_2_ concentration positively impacted photosynthesis in sweet sorghum, resulting in a 5.8% increase in *A*_nmax_ (**Table 5**, **Supplementary Table 4**). Similar decreases occurred in *g*_s-max_ and *T*_r-max_, but the plants’ dependence on light increased, as evidenced by *I*_sat_, *I*_g-sat_ and *I*_T-sat_, all exceeding 2000 μmol photons m^–2^ s^–1^. Notably, WUE_i-max_ showed significant improvement, reaching 255.46 ± 5.26 μmol mol^–1^, significantly higher than all other treatment groups (one-way and two-way ANOVA *p*<0.05) (**Tables 4**, **5**). However, the increase in WUE_inst-max_ was significantly lower compared to the increase observed under elevated CO_2_ at 30°C, reaching only 37.45% (**Supplementary Table 4**).

### Interactive effect of temperature and CO_2_ concentration on key photosynthetic parameters

The interactive effect of temperature and CO_2_ concentration on key photosynthetic parameters was assessed in sweet sorghum using a two-way analysis of variance (ANOVA) (**Table 4**). Temperature provoked highly significant main effects (*p*<0.001) on *A*_nmax_, *I*_sat_, *I*_c_, *R*_d_, *g*_s-max_, *T*_r-max_, and WUE_inst-max_, suggesting increasing the temperature from 30°C to 35°C profoundly impacted these traits independently of CO_2_ concentration. Meanwhile, elevated CO_2_ concentration induced highly significant main effect (*p*<0.001) on *I*_sat_, *g*_s-max_, *T*_r-max_, and WUE_i-max_, regardless of temperature. Statistically significant interaction effects between temperature and CO_2_ were observed for the intrinsic quantum yield (*α*) at *p*<0.05, as well as for WUE_i-max_, and WUE_inst-max_. Quantum yield represents the photon requirement for assimilating one CO_2_ molecule during photosynthesis. This suggests that the impact of temperature differed across CO_2_ levels in determining the ability of sweet sorghum to acquire and use water.

## DISCUSSION

### Applicability of Ye's models to NRH model

In this study, we employed a methodological approach that involved a comparative analysis of models against observed data. This facilitated a systematic assessment and differentiation of the Ye's models and the NRH model concerning their abilities to replicate the *A*_n_-*I*, *g*_s_-*I*, *T*_r_-*I*, WUE_i_-*I*, and WUE_inst_-*I* curves of sweet sorghum. We also focused on quantifying the key traits defining these curves. Remarkably, the Ye's models excelled not only in capturing the diminishing trends in photosynthetic parameters (e.g. *A*_n_, WUE_i_, WUE_inst_) with increased light intensity, but also in accurately determining critical photosynthetic traits across diverse temperatures and CO_2_ conditions. These findings align with previous research applying the Ye's models to Cyanobacteria (Yang et al., 2022; Yang et al., 2023), Chlorophyta, Bacillariophyta (Yang et al., 2020), and other plants (Xu et al., 2012; Atsushi et al., 2017; Martínez-García et al., 2017; Liu et al., 2018; Ye et al., 2020), demonstrating its broad utility.

Since its establishment by Thornley in 1976, the NRH model has been extensively utilized to study the response of plant photosynthesis to light, elucidating changes in plant growth and physiology across diverse environments (Yu et al., 2004; Ye and Yu, 2008; dos Santos Junior et al., 2012; Xu et al., 2012; Domingues et al., 2014; Wassenaar et al., 2022; Wu et al., 2023). However, upon taking the first derivative of the mathematical expression characterizing the relationship between the variables *A*_n_ and *I*, as defined by Equation 13 in the NRH model, an analytical solution was not obtained. This implies that the NRH model follows an asymptotic function for *A*_n_ rather than a closed-form solution. Consequently, the NRH model does not produce analytical solutions for the maximum *A*_n_ and the specific saturation light intensity for *A*_nmax_. *A*_nmax_ may be numerically estimated from the NRH model through nonlinear least squares calculations (Ye et al., 2021). When parameters such as *A*_n_, *g*_s_, *T*_r_, WUE_i_, and WUE_inst_ of plants reach saturation or decrease with increasing *I* under high light intensities, the maximum values obtained by fitting their light response curves using the NRH model would inevitably deviate significantly from their observed values. This discrepancy explains why the *A*_nmax_, *g*_s-max_, *T*_r-max_, WUE_i-max_, and WUE_inst-max_ derived from the NRH model fitting for sweet sorghum in this study significantly differed from the measured data. These findings are consistent with previous reports of similar discrepancies (dos Santos Junior et al., 2012; Xu et al., 2012; Atsushi et al., 2017; Martínez-García et al., 2017; Liu et al., 2018).

Photoinhibition is the well-documented, light-induced decrease in photosynthetic rate in plants that occurs upon exposure to excess irradiance exceeding their utilization capacity (Anderson et al., 2021). Similarly, in the present study, both the WUE_i_-*I* and WUE_inst_-*I* curves of sweet sorghum exhibited declining trends under high light intensities, consistent with our previous findings in *Glycine max* and *Amaranthus hypochondriacus* (Ye et al., 2020). This reduction in plant WUE under high irradiance appears to be common across species. Thus, light response models that do not effectively capture decreases in photosynthetic parameters under high light conditions are likely to fail in elucidate plant responses across varying irradiance environments and determine actual physiological states (Egea et al., 2011). In contrast, by incorporating light inhibition effect (*β*) and a light saturation parameter (*γ*), the Ye's model (Equations 1, 7, 10 ) provided excellent fits to the *A*_n_-*I*, WUE_i-max_-*I*, and WUE_inst-max_-*I* curves of plants, irrespective of temperature or CO_2_ concentration (Ye et al., 2013; Ye et al., 2020).

### Effect of elevated temperature on dynamic photosynthesis and water use efficiency in sweet Sorghum leaves

Plants facing environmental stress commonly exhibit reduced leaf photosynthesis (Hatfield and Dold, 2019). However, our study revealed sweet sorghum exhibited robust photosynthesis and a lack of photoinhibition at high temperatures up to 35°C, aligning with literature suggesting greater photosynthetic thermotolerance in sorghum species (Loreto et al., 1995; Prasad et al., 2021; Sales et al., 2021). Specifically, previous evidence indicates that optimal temperatures exceeding 35°C sustain the enzymatic rates of key C_4_ cycle enzymes like pyruvate orthophosphate dikinase and phosphoenolpyruvate carboxylase in sorghum (Loreto et al., 1995; Sales et al., 2021), thereby preserving carbon assimilation even at high temperatures. Through concentrating CO_2_ at the site of Rubisco, C_4_ plants effectively suppress photorespiration while simultaneously minimizing transpiration. This twin benefit confers advantages to C_4_ species over C_3_ plants in hot, arid climates (Sales et al., 2021). Therefore, the thermotolerant photosynthesis exhibited by sweet sorghum likely facilitates the ecological success of C_4_ cereals including sorghum across tropical and subtropical agroecosystems prone to heat stress. Further examination of genotypic variation in thermal acclimation processes governing photosynthesis may inform climate-resilient crop development.

*g*_s_ has been considered one of the most vital indicators of a plant’s response to environmental fluctuations, yet its reaction to temperature has been relatively understudied (Urban et al., 2017). Some earlier studies have suggested divergent responses of *g*_s_ to increasing temperatures, including an increase (Mott and Peak, 2010; Urban et al., 2017), no significant change, or even stomatal closure. Our present results are consistent with previous research in *Tradescantia pallida* (Mott and Peak, 2010), *Pinus taeda*, and *Populus deltoides x nigra* (Urban et al., 2017). As temperatures increased, *g*_s_ of sweet sorghum gradually increased, enhancing the exchange of CO_2_ and H_2_O and elevating the CO_2_ uptake. In early research, *g*_s_ and *A*_n_ were commonly presumed to share a stable, linear relationship, with *g*_s_ and *A*_n_ exhibiting synergistic increases or decreases in response to varying environmental conditions. The patterns of *g*_s_ and *A*_n_ in sweet sorghum leaves showed some parallels when responding to light. However, because *g*_s_ is more thermally sensitive, a slight decline in WUE_i_ was observed when leaf temperatures increased from 30°C to 35°C. Typically, increased temperature coupled with heightened vapor pressure deficit (*VPD*) results in increased *T*_r_, followed by cell shrinkage and stomatal closure (Urban et al., 2017; Sales et al., 2021). Interestingly in this study, even when the *VPD* of sweet sorghum leaves rose to 2.2 kPa at 35°C, the intercellular CO_2_ concentration (*C*_i_) remained relatively stable, implying stomata did not close. Instead, *VPD*, *g*_s_, and *A*_n_ increased concurrently (**Supplementary Figure 2**, **Figures 1A-F**). This aligns with observations in *Oryza* and *Flaveria bidentis* under elevated temperatures (Kubien et al., 2003; Scafaro et al., 2011), contrasting the behavior of the C_4_ Model *Setaria viridis* (Anderson et al., 2021). The differential stomatal responses to warming may be attributable to variances in plant functional type or evolutionary origin (Bykova et al., 2019). Therefore, the findings indicate photosynthesis in sweet sorghum was not suppressed by elevated temperatures; rather, it was stimulated by widening of stomatal openings to enhance CO_2_ influx, expediting assimilation rates.

The variation in WUE_inst_ of sweet sorghum became more noticeable with increasing temperature. Notably, WUE_inst_ substantially decreased relative to WUE_i_, primarily due to the disproportionate increase in transpiration compared to photosynthesis under elevated temperatures. For example, from 30°C to 35°C, *T*_r-max_ increased 2.73 times compared to *A*_nmax_ and 2.02 times versus *g*_s-max_. WUE_i_ indicates a long-term water use balance between assimilation and conductance, independent of vapor pressure deficit (von Caemmerer and Farquhar, 1981; Gyimah and Nakao, 2007; Ye et al., 2020). In this study, sweet sorghum exhibited a minor decrease in WUE_i_ with elevated temperatures, highlighting adaptation via prioritizing growth and survival over short-term heat stress. In contrast, WUE_inst_ experienced a significant decrease. As WUE_inst_ represents plant yield per water loss, aligning with whole-plant WUE (Wall et al., 2001). Its substantial reduction indicates a trade-off of water to ensure survival under high temperatures. This could be seen as a physiological mechanism employed by sweet sorghum for leaf cooling under short-term heat stress, as adopted in other species (Scafaro et al., 2011; Anderson et al., 2021). Collectively, these results reveal that sweet sorghum adjustments to maintain photosynthesis, transpiration and respiration could outweigh slower WUE adaptations, especially diminishing dynamic WUE_inst_.

### Effect of elevated CO_2_ concentration on dynamic photosynthesis and water use efficiency in sweet sorghum leaves

Raising CO_2_ levels from 250 to 410 μmol mol^–1^ enhanced photosynthesis in sweet sorghum at moderate temperatures, consistent with observations in many plants (Gago et al., 2014; Kimball, 2016). The heightened substrate availability drives this initial photosynthetic response. However, further elevating CO_2_ concentration to 550 μmol mol^–1^ did not lead to a consistent and proportional increase in photosynthesis. In concordance with previous research indicating reduced sorghum yield under elevated CO_2_ concentrations (Kimball, 2016; Asadi and Eshghizadeh, 2021), this suggests that sweet sorghum’s photosynthetic system cannot fully capitalize on CO_2_ levels beyond a saturation threshold. Instead, alternative factors such as light availability, may become limiting beyond this point. Despite lacking statistical significance, elevated CO_2_ concentration did improve photosynthesis at 35°C. Two-way ANOVA analysis from **Table 4** also indicated CO_2_ level impacts on sweet sorghum’s light use efficiency and WUE (including both intrinsic and instantaneous aspects) vary across temperatures, highlighting complex interplay between temperature and CO_2_ in photosynthetic responses. Previous research emphasized that the magnitude of *A*_n_ at elevated temperatures is primarily determined by the electron transfer capacity, suggesting that sweet sorghum photosynthesis has been constrained by irradiance and temperature under current atmospheric CO_2_ (Loreto et al., 1995; McAusland et al., 2016; Sales et al., 2021).

The CO_2_ rise induced partial stomatal closure, reducing *g*_s_ and *T*_r_, consequently improving WUE, a widely observed response (Conley et al., 2001; Allen et al., 2011). The reduction in *g*_s_ under high CO_2_ levels is recognized as an adaptive response across various plant species (Farooq et al., 2019). Higher atmospheric CO_2_ led to elevated *C*_i_ in leaves, increasing stomatal sensitivity and prompting adjustments to maintain *C*_i_ below ambient levels (Urban et al., 2017; Feng et al., 2023). Contrary to previous studies attributing heightened WUE_i_ to simultaneous increases in *A*_n_ and decreases in *T*_r_ under elevated CO_2_ (Conley et al., 2001; Ye et al., 2020), improvements here arose primarily from reductions in *g*_s_ and *T*_r_, rather than an increase in *A*_n_. Furthermore, CO_2_-induced reductions in *g*_s_ and *T*_r_ diminished at 35°C, suggesting that this stomatal response weakens with increasing leaf temperature. This interaction emphasizes the complex CO_2_-temperature interplay in shaping plant responses. Despite the substantial increase in WUE_i_ to 255.46 ± 5.26 μmol mol^–1^ under the combined influence of elevated CO_2_ and temperature implies the adaptive capacity of sweet sorghum to shifting environments, the precise mechanisms remain unclear and require further investigation. Optimizing WUE likely involves intricate signaling and physiological adjustments (Hatfield and Dold, 2019).

While providing unique insights into immediate photosynthetic responses, our short-term exposures are constrained in comparison to long-term open-top chamber (OTC) and free-air CO_2_ enrichment (FACE) studies. Prolonged OTC and FACE exposures over weeks or months facilitate a thorough characterization of gradual acclimation processes, revealing cumulative impacts on physiology, metabolism, tissue structure, gene/protein expression, yield, and overall plant adaptation. Looking ahead, it is imperative to conduct systematic and long-term investigations to gain a more nuanced understanding of sweet sorghum’s adaptability to climate change scenarios and its potential under predicted climatic conditions. These future studies should meticulously explore morphological, physiological, and biochemical differences within the plant, encompassing variations in responses among roots, stems, and leaves. Additionally, our use of indoor-cultivated seedlings differs from field-grown plants. While growth chambers allow precise regulation of conditions like temperature, humidity, and lighting to reduce variability and stressors, indoor cultivation may heighten sensitivity to environmental shifts. Transferring seedlings from controlled growth chamber to the lab with open gas circulation for gas exchange measurements may induce physiological changes due to altered growth conditions. Despite efforts to minimize variation, the transition between environments could result in transient responses that would not occur in plants already acclimated to field settings.

## Conclusion

Our study further validates the superiority of the photosynthesis models proposed by Ye et al. for simulating the light response curves of *A*_n_, *g*_s_, *T*_r_, WUE_i_, and WUE_inst_. Particularly, when employed to fit the WUE_i_-*I* and WUE_inst_-*I* curves, the models uniquely capture the decline in both WUE_i_ and WUE_inst_ with increasing *I* under high light intensity. Across all experimental conditions, it returns the values for *A*_nmax_, *g*_s-max_, *T*_r-max_, WUE_i-max_, and WUE_inst-max_ closely matching measured data. In contrast, the NRH model inadequately reproduces the WUE_i_-*I* and WUE_inst_-*I* curves and severely overestimates *A*_nmax_, WUE_i-max_, and WUE_inst-max_ relative to measurements. It also fails to determine the corresponding saturation light intensities for these traits. In addition, sweet sorghum exhibits remarkable heat tolerance up to 35°C, achieving higher *A*_n_ through stimulated *g*_s_. However, disproportionate increase in *T*_r_ lead to a sharp decline in WUE_inst_. Increasing CO_2_ concentration from sub-ambient to ambient levels has a significant positive effect on photosynthesis in sweet sorghum, but no consistent and proportional increase is observed at 550 μmol mol^−1^. Elevated CO_2_ causes partial stomatal closure in sweet sorghum leaves, markedly reducing *g*_s_ and *T*_r_, thereby improving WUE_i_ and WUE_inst_. Nevertheless, the effect of CO_2_-induced stomatal closure in reducing transpiration progressively diminishes at high temperature owing to stomatal opening. Elucidating the physiological mechanisms governing plant responses to combined environmental factors will enable identifying adaptive traits to accelerate resilient crop development amidst intensifying environments.

## Data availability statement

The original contributions presented in the study are included in the article/**Supplementary Material**. Further inquiries can be directed to the corresponding authors.

## Author contributions

X-LY: Conceptualization, Data curation, Formal Analysis, Investigation, Writing – original draft. X-FM: Writing – review and editing, Resources, Supervision, Software. Z-PY: Funding acquisition, Methodology, Writing – review and editing, Software. L-SY: Writing – review and editing, Investigation. J-BS: Writing – review and editing, Investigation. XW: Writing – review and editing, Investigation. B-BZ: Writing – review and editing, Visualization. F-BW: Writing – review and editing. Z-FD: Funding acquisition, Supervision, Writing – review and editing, Project administration.
