# Supplementary material for: Simulating short-term light responses of photosynthesis and water use efficiency in sweet sorghum under varying temperature and CO2 conditions
Source: Front Plant Sci. 2024 Mar 28;15:1291630. doi: 10.3389/fpls.2024.1291630 (PMC11007071; doi:10.3389/fpls.2024.1291630)
Supplement: Supplementary file 1 [file DataSheet_1.doc]

**Simulating short-term Light Responses of Photosynthesis and Water Use Efficiency in Sweet Sorghum under Varying Temperature and CO_2_ Conditions**

*Xiao-Long, Yang^1,2^; Xiao-Fei, Ma^3^; Zi-Piao, Ye^4^*; Long-Sheng Yang^1^; Jun-Bo Shi^1^; Xun Wang^1^**; Bei-Bei Zhou^1^; Fu-Biao, Wang^4^; Zi-Fa, Deng^1^**

*^1^School of Life Sciences, Nantong University, Nantong, China,* *^2^State Key Laboratory of Environmental Chemistry and Ecotoxicology, Research Center for Eco-Environmental Sciences, Chinese Academy of Sciences,* *Beijing, China, ^3^Key Laboratory of Ecological Safety and Sustainable Development in Arid Lands, Northwest Institute of Eco-Environment and Resources, Chinese Academy of Sciences, Lanzhou, China, ^4^Institute of Biophysics in Maths & Physics College, Jinggangshan University, Ji’an, China*

** For Correspondence:*

*Zi-Piao, Ye, E-mail: yezp@jgsu.edu.cn; Zi-Fa, Deng, E-mail: dzf@ntu.edu.cn*


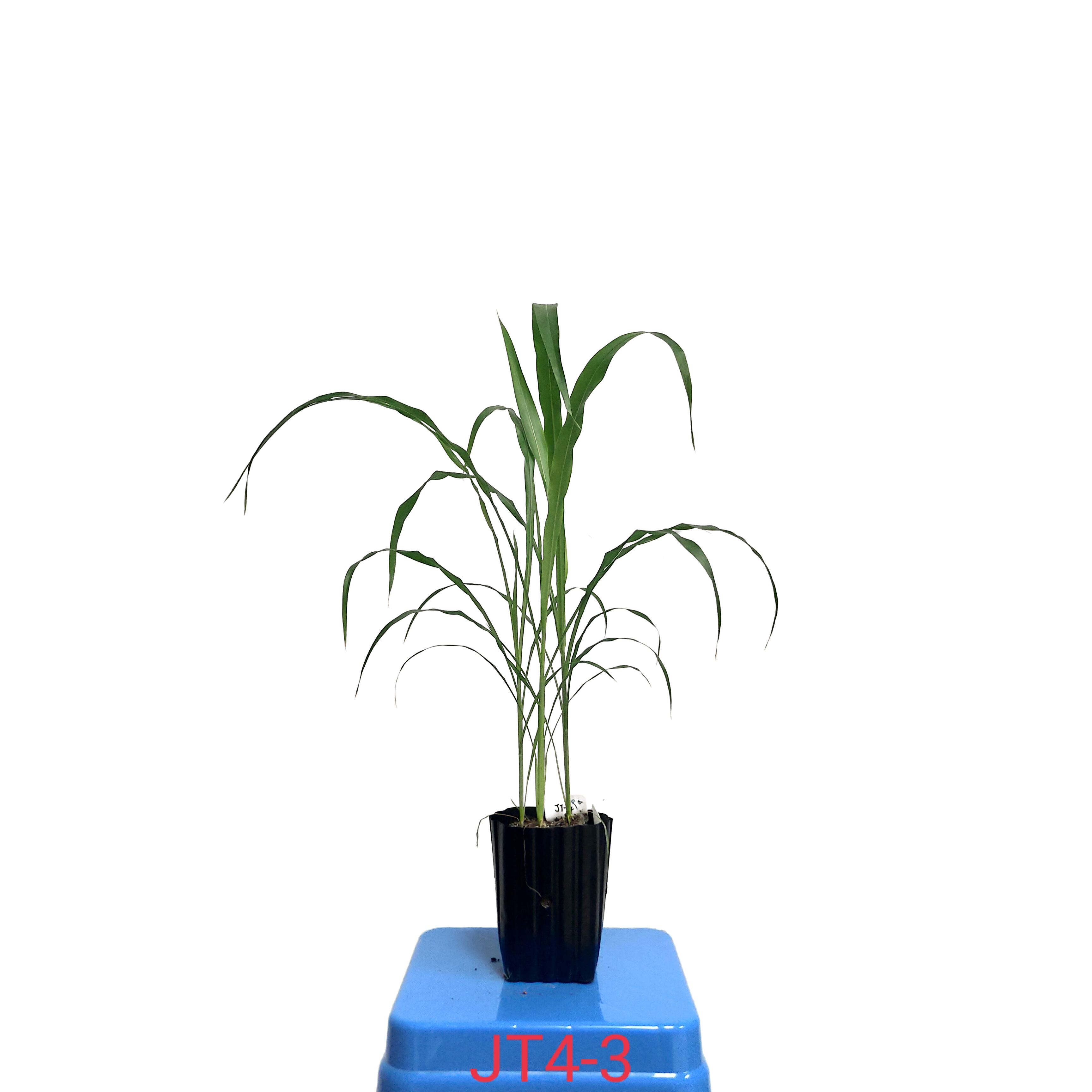


**FIGURE S1 | Growth performance of sweet sorghum KFJT-4 after cultivation of 40 days**

**TABLE S1 | Growth traits of sweet sorghum KFJT-4 after cultivation of 40 days. n is the number of plants.**

| **Parameters** | **Measured values (mean** ± ***SE*, *n* = 23)** |
| --- | --- |
| Natural plant height (cm) | 62.96 ± 2.54 |
| Banded plant height (cm) | 89.39 ± 3.32 |
| Stem diameter (cm) | 7.75 ± 0.38 |
| Number of leaves | 7.30 ± 0.23 |
| Leaf area (cm^2^) | 103.40 ± 8.53 |
| Leaf length (cm) | 52.71 ± 2.61 |
| Average width (cm) | 1.85 ± 0.09 |
| Maximum width (cm) | 2.64 ± 0.10 |

**TABLE S2 | Fitted (Ye model and NRH model) and measured values of maximum net photosynthetic rate (*A*_nmax_), saturation irradiance corresponding to *A*_nmax_ (*I*_sat_), light-saturated stomatal conductance (*g*_s-max_), saturation irradiance corresponding to *g*_s-max_ (*I*_g-sat_), light-saturated transpiration rate *(T*_r-max_), saturation irradiance corresponding to *T*_r-max_ (*I*_T-sat_), maximum intrinsic water use efficiency (WUE_i_), saturation irradiance corresponding to WUE_i_ (*I*_i-sat_), maximum instantaneous water use efficiency (WUE_inst-max_), and saturation irradiance corresponding to WUE_inst-max_ (*I*_inst-sat_) for sweet sorghum under different temperature conditions at atmospheric CO_2_ level.**

| **T**  **(℃)** | ***A*_nmax_ (μmol m^–2^ s^–1^)** | | |  | ***I*_sat_ (μmol m^–2^ s^–1^)** | | |
| --- | --- | --- | --- | --- | --- | --- | --- |
|  | **Ye model** | **NRH model** | **Measured value** |  | **Ye model** | **NRH model** | **Measured value** |
| 25 | 20.70 ± 0.73 ^b^ | 21.07 ± 1.02 ^a^ | 20.13 ± 0.68 ^a^ |  | 1434.9 ± 92.9 ^a^ | ― | 1400.0 ± 115.5 ^a^ |
| 30 | 30.54 ± 0.76 ^a^ | 32.25 ± 1.68 ^a^ | 29.96 ± 1.47 ^a^ |  | 1460.8 ± 169.7 ^a^ | ― | 1466.7 ± 66.7 ^a^ |
| 35 | 37.67 ± 0.96 ^b^ | 42.30 ± 1.21 ^a^ | 37.49 ± 0.90 ^a^ |  | 1710.8 ± 31.3 ^b^ | ― | 1866.7 ± 33.3 ^a^ |
|  | ***g*_s-max_ (mol m^–2^ s^–1^)** | | |  | ***I*_g-sat_ (μmol m^–2^ s^–1^)** | | |
|  | **Ye model** | **NRH model** | **Measured value** |  | **Ye model** | **NRH model** | **Measured value** |
| 25 | 0.138 ± 0.012 ^a^ | 0.123 ± 0.012 ^a^ | 0.137 ± 0.012 ^a^ |  | 1765.9 ± 158.0 ^a^ | ― | 1800.0 ± 115.5 ^a^ |
| 30 | 0.194 ± 0.010 ^a^ | 0.167 ± 0.011 ^a^ | 0.191 ± 0.010 ^a^ |  | 1654.9 ± 22.3 ^a^ | ― | 1600.0 ± 0.0 ^a^ |
| 35 | 0.252 ± 0.009 ^a^ | 0.259 ± 0.004 ^a^ | 0.257 ± 0.008 ^a^ |  | 1900.9 ± 74.5 ^a^ | ― | 2000.0 ± 0.0 ^a^ |
|  | ***T*_r-max_ (mmol m^–2^ s^–1^)** | | |  | ***I*_T-sat_ (μmol m^–2^ s^–1^)** | | |
|  | **Ye model** | **NRH model** | **Measured value** |  | **Ye model** | **NRH model** | **Measured value** |
| 25 | 1.68 ± 0.11 ^a^ | 1.54 ± 0.11 ^a^ | 1.72 ± 0.11 ^a^ |  | 2292.3 ± 392.2 ^a^ | ― | 2000 ^a^ |
| 30 | 3.56 ± 0.15 ^a^ | 4.01 ± 0.48 ^a^ | 3.58 ± 0.20 ^a^ |  | 2578.4 ± 319.2 | ― | >2000 |
| 35 | 5.83 ± 0.27 ^a^ | 6.52 ± 0.26 ^a^ | 5.76 ± 0.21 ^a^ |  | 2663.2 ± 310.5 | ― | >2000 |
|  | **WUE_i-max_ (μmol mol^–1^)** | | |  | ***I*_i-sat_ (μmol m^–2^ s^–1^)** | | |
|  | **Ye model** | **NRH model** | **Measured value** |  | **Ye model** | **NRH model** | **Measured value** |
| 25 | 174.2 ± 3.6 ^b^ | 200.2 ± 9.8 ^a^ | 174.3 ± 5.2 ^b^ |  | 615.4 ± 32.2 ^a^ | ― | 466.7 ± 176.4 ^a^ |
| 30 | 163.1 ± 2.1 ^b^ | 192.5 ± 8.9 ^a^ | 161.4 ± 2.4 ^b^ |  | 965.4 ± 76.8 ^a^ | ― | 733.3 ± 66.7 ^a^ |
| 35 | 151.4 ± 6.5 ^b^ | 225.7 ± 18.6 ^a^ | 167.4 ± 18.1 ^b^ |  | 859.9 ± 222.5 ^a^ | ― | 733.3 ± 33.3 ^a^ |
|  | **WUE_inst-max_ (μmol mmol^–1^)** | | |  | ***I*_inst-sat_ (μmol m^–2^ s^–1^)** | | |
|  | **Ye model** | **NRH model** | **Measured value** |  | **Ye model** | **NRH model** | **Measured value** |
| 25 | 14.12 ± 0.22 ^b^ | 16.03 ± 0.64 ^a^ | 13.81 ± 0.28 ^b^ |  | 602.7 ± 18.0 ^a^ | ― | 633.3 ± 120.2 ^a^ |
| 30 | 9.70 ± 0.20 ^b^ | 10.94 ± 0.58 ^a^ | 9.71 ± 0.18 ^b^ |  | 799.1 ± 63.6 ^a^ | ― | 666.7 ± 88.2 ^a^ |
| 35 | 7.21 ± 0.35 ^b^ | 10.45 ± 0.89 ^a^ | 8.07 ± 0.87 ^b^ |  | 728.2 ± 130.1 ^a^ | ― | 600.0 ± 115.5 ^a^ |

*At a given temperature, different letters denote statistically significant differences (p<0.05) among the measured value, the value fitted by the Ye model and the value fitted by the NRH model.*

**TABLE S3 | Fitted (Ye model and NRH model) and measured values of maximum net photosynthetic rate (*A*_nmax_), saturation irradiance corresponding to *A*_nmax_ (*I*_sat_), light-saturated stomatal conductance (*g*_s-max_), saturation irradiance corresponding to *g*_s-max_ (*I*_g-sat_), light-saturated transpiration rate *(T*_r-max_), saturation irradiance corresponding to *T*_r-max_ (*I*_T-sat_), maximum intrinsic water use efficiency (WUE_i_), saturation irradiance corresponding to WUE_i_ (*I*_i-sat_), maximum instantaneous water use efficiency (WUE_inst-max_), and saturation irradiance corresponding to WUE_inst-max_ (*I*_inst-sat_) for sweet sorghum under different CO_2_ concentrations at 30℃.**

| **CO_2_ level**  **(μmol mol^–1^)** | ***A*_nmax_ (μmol m^–2^ s^–1^)** | | |  | ***I*_sat_ (μmol m^–2^ s^–1^)** | | |
| --- | --- | --- | --- | --- | --- | --- | --- |
|  | **Ye model** | **NRH model** | **Measured value** |  | **Ye model** | **NRH model** | **Measured value** |
| 250 | 25.84 ± 0.65 ^b^ | 27.01 ± 0.71 ^b^ | 25.22 ± 0.617 ^b^ |  | 1393.8 ± 71.5 ^a^ | ― | 1466.7 ± 133.3 ^a^ |
| 410 | 30.54 ± 0.76 ^a^ | 32.25 ± 1.68 ^a^ | 29.96 ± 1.47 ^a^ |  | 1460.8 ± 169.7 ^a^ | ― | 1466.7 ± 66.7 ^a^ |
| 550 | 29.88 ± 1.07 ^b^ | 33.43 ± 1.10 ^a^ | 30.17 ± 0.88 ^b^ |  | 1782.4 ± 30.1 ^a^ | ― | 1866.7 ± 66.7 ^a^ |
|  | ***g*_s-max_ (mol m^–2^ s^–1^)** | | |  | ***I*_g-sat_ (μmol m^–2^ s^–1^)** | | |
|  | **Ye model** | **NRH model** | **Measured value** |  | **Ye model** | **NRH model** | **Measured value** |
| 250 | 0.293 ± 0.012 ^a^ | 0.237 ± 0.040 ^a^ | 0.291 ± 0.012 ^a^ |  | 1484.6 ± 184.4 ^a^ | ― | 1533.3 ± 66.7 ^a^ |
| 410 | 0.194 ± 0.010 ^a^ | 0.167 ± 0.011 ^a^ | 0.191 ± 0.010 ^a^ |  | 1654.9 ± 22.3 ^a^ | ― | 1600.0 ± 0.0 ^a^ |
| 550 | 0.156 ± 0.008 ^a^ | 0.161 ± 0.002 ^a^ | 0.156 ± 0.010 ^a^ |  | 2533.6 ± 328.5 | ― | >2000 |
|  | ***T*_r-max_ (mmol m^–2^ s^–1^)** | | |  | ***I*_T-sat_ (μmol m^–2^ s^–1^)** | | |
|  | **Ye model** | **NRH model** | **Measured value** |  | **Ye model** | **NRH model** | **Measured value** |
| 250 | 5.48 ± 0.36 ^a^ | 5.13 ± 0.76 ^a^ | 5.59 ± 0.53 ^b^ |  | 2378.8 ± 142.1 | ― | >2000 |
| 410 | 3.56 ± 0.15 ^a^ | 4.01 ± 0.48 ^a^ | 3.58 ± 0.20 ^a^ |  | 2578.4 ± 319.2 | ― | >2000 |
| 550 | 2.62 ± 0.13 ^a^ | 2.74 ± 0.04 ^a^ | 2.58 ± 0.19 ^a^ |  | 2837.3 ± 396.3 | ― | >2000 |
|  | **WUE_i-max_ (μmol mol^–1^)** | | |  | ***I*_i-sat_ (μmol m^–2^ s^–1^)** | | |
|  | **Ye model** | **NRH model** | **Measured value** |  | **Ye model** | **NRH model** | **Measured value** |
| 250 | 88.6 ± 2.7 ^b^ | 120.8 ± 13.7 ^a^ | 88.70 ± 1.70 ^b^ |  | 1227.7 ± 62.6 ^a^ | ― | 1133.3 ± 133.3 ^a^ |
| 410 | 163.1 ± 2.1 ^b^ | 192.5 ± 8.9 ^a^ | 161.4 ± 2.4 ^b^ |  | 965.4 ± 76.8 ^a^ | ― | 733.3 ± 66.7 ^a^ |
| 550 | 232.4 ± 6.6 ^b^ | 281.7 ± 12.9 ^a^ | 237.2 ± 8.9 ^b^ |  | 806.8 ± 54.0 ^a^ | ― | 733.3 ± 66.7 ^a^ |
|  | **WUE_inst-max_ (μmol mmol^–1^)** | | |  | ***I*_inst-sat_ (μmol m^–2^ s^–1^)** | | |
|  | **Ye model** | **NRH model** | **Measured value** |  | **Ye model** | **NRH model** | **Measured value** |
| 250 | 5.26 ± 0.19 ^b^ | 7.65 ± 0.94 ^a^ | 5.27 ± 0.15 ^b^ |  | 985.7 ± 14.9 ^a^ | ― | 933.3 ± 66.7 ^a^ |
| 410 | 9.70 ± 0.20 ^b^ | 10.94 ± 0.58 ^a^ | 9.71 ± 0.18 ^b^ |  | 799.1 ± 63.6 ^a^ | ― | 666.7 ± 88.2 ^a^ |
| 550 | 14.05 ± 0.47 ^b^ | 16.95 ± 0.91 ^a^ | 14.30 ± 0.54 ^b^ |  | 815.7 ± 49.9 ^a^ | ― | 733.3 ± 66.7 ^a^ |

*At a given CO_2_ concentrations, different letters denote statistically significant differences (p<0.05) among the measured value, the value fitted by the Ye model and the value fitted by the NRH model.*

**TABLE S4 | Interactive effects of elevating CO_2_ and warming on photosynthesis of sweet sorghum.**

| **Photosynthetic parameters** | **35 ℃** | **30 ℃** | **35 ℃** |
| --- | --- | --- | --- |
|  | **410 μmol mol^–1^** | **550 μmol mol^–1^** | **550 μmol mol^–1^** |
| *α* | 0.0732 ± 0.0026 ^a^ | 0.0610 ± 0.0068 ^b^ | 0.0649 ± 0.0033 ^ab^ |
| *A*_nmax_ (μmol m^–2^ s^–1^) | 37.67 ± 0.96 ^a^ | 29.88 ± 1.07 ^b^ | 39.87 ± 1.92 ^a^ |
| *I*_sat_ (μmol m^–2^ s^–1^) | 1710.8 ± 31.3 ^b^ | 1782.36 ± 30.13 ^b^ | 2064.90 ± 45.82 ^a^ |
| *I*_c_ (μmol m^–2^ s^–1^) | 39.27 ± 5.19 ^a^ | 28.87 ± 1.90 ^b^ | 38.64 ± 2.9 ^a^ |
| *R*_d_ (μmol m^–2^ s^–1^) | 2.80 ± 0.44 ^a^ | 1.70 ± 0.13 ^b^ | 2.43 ± 0.18 ^a^ |
| *α*_s_ | 0.0004 ± 0.0000 ^a^ | 0.0002 ± 0.0000 ^b^ | 0.0002 ± 0.0000 ^b^ |
| *g*_s-max_ (mol m^–2^ s^–1^) | 0.252 ± 0.008 ^a^ | 0.156 ± 0.008 ^c^ | 0.215 ± 0.01 ^b^ |
| *I*_g-sat_ (μmol m^–2^ s^–1^) | 1900.89 ± 74.45 ^b^ | 2533.57 ± 328.53 ^b^ | 3484.01 ± 395.74 ^a^ |
| *g*_s0_ (mol m^–2^ s^–1^) | 0.011 ± 0.008 ^a^ | 0.016 ± 0.001 ^a^ | 0.006 ± 0.003 ^a^ |
| *α*_r_ | 0.0072 ± 0.0006 ^a^ | 0.0028 ± 0.0003 ^c^ | 0.0053 ± 0.0007 ^b^ |
| *T*_r-max_ (mmol m^–2^ s^–1^) | 5.83 ± 0.27 ^a^ | 2.62 ± 0.13 ^b^ | 4.94 ± 0.45 ^a^ |
| *I*_T-sat_ (μmol m^–2^ s^–1^) | 2663.16 ± 310.52 ^a^ | 2837.28 ± 396.25 ^a^ | 2561.44 ± 123.51 ^a^ |
| *T*_r0_ (mmol m^–2^ s^–1^) | 0.25 ± 0.16 ^a^ | 0.27 ± 0.02 ^a^ | 0.21 ± 0.06 ^a^ |
| *α*_i_ | 3.9871 ± 1.412 ^b^ | 3.1903 ± 0.5037 ^b^ | 9.1759 ± 1.8803 ^a^ |
| WUE_i-max_ (μmol mol^–1^) | 151.34 ± 6.46 ^c^ | 232.37 ± 6.57 ^b^ | 255.46 ± 5.26 ^a^ |
| *I*_i-sat_ (μmol m^–2^ s^–1^) | 859.94 ± 222.47 ^a^ | 806.83 ± 54.02 ^ab^ | 644.44 ± 49.65 ^b^ |
| *K*_i_ (μmol mol^–1^) | 75.18 ± 19.12 ^b^ | 66.68 ± 7.90 ^b^ | 181.31 ± 25.48 ^a^ |
| *α*_inst_ | 0.1706 ± 0.0618 ^a^ | 0.1849 ± 0.0293 ^a^ | 0.2941 ± 0.0656 ^a^ |
| WUE_inst-max_ (μmol mol^–1^) | 7.21 ± 0.35 ^c^ | 14.05 ± 0.47 ^a^ | 9.91 ± 0.64 ^b^ |
| *I*_inst-sat_ (μmol m^–2^ s^–1^) | 728.23 ± 30.09 ^a^ | 815.71 ± 49.94 ^a^ | 816.50 ± 54.83 ^a^ |
| *K*_inst_ (μmol mol^–1^) | 3.43 ± 0.88 ^b^ | 3.91 ± 0.47 ^b^ | 6.19 ± 0.99 ^a^ |

*Different letters denote statistically significant differences (p<0.05) among the values fitted by the Ye model, the values fitted by the NRH model, and the measured values*.


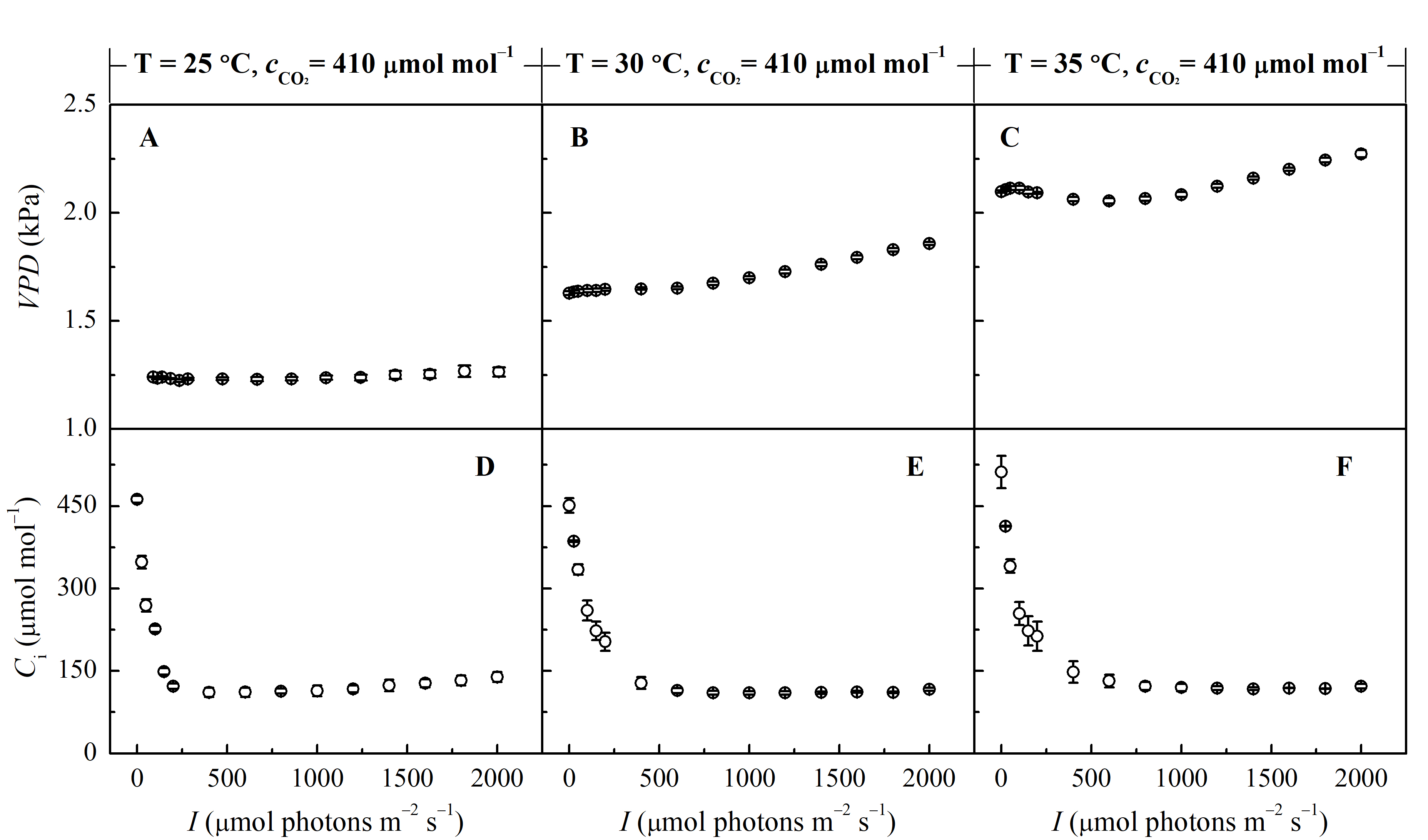


**FIGURE S2 | Vapor pressure deficit (VPD) (A, B, C) and intercellular CO_2_ concentration (*C*_i_) (D, E, F) over irradiance (*I*) levels for sweet sorghum under different temperature conditions at atmospheric CO_2_ level.**


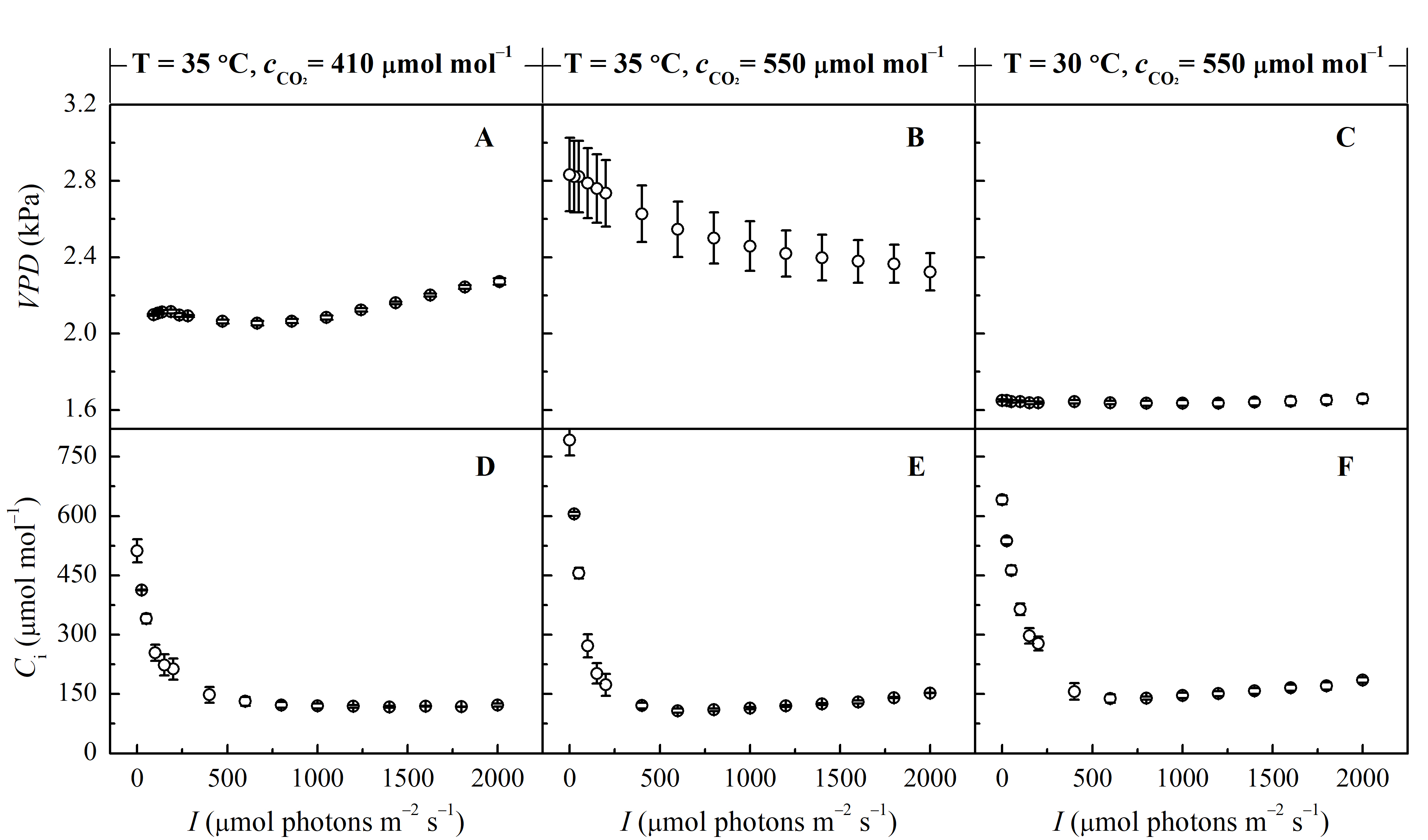


**FIGURE S3 | Vapor pressure deficit (VPD) (A, B, C) and intercellular CO_2_ concentration (*C*_i_) (D, E, F) over irradiance (*I*) levels for sweet sorghum under different temperature and CO_2_ concentration conditions.**
